# A transcriptional atlas of gyrification reveals dynamic spatio-temporal gene expression in the developing ferret cortex

**DOI:** 10.1007/s00429-026-03178-6

**Published:** 2026-08-12

**Authors:** Mikaela Barresi, Alice Johnstone, Sebastian Quezada, Anita Quigley, Mary Tolcos

**Affiliations:** 1 School of Health and Biomedical Sciences, RMIT University, Building 223, 225-245 Plenty Rd, Bundoora, VIC 3083 Australia; 2 School of Science, RMIT University, Melbourne, VIC 3083 Australia; 3 Aikenhead Centre for Medical Discovery (ACMD), St Vincent’s Hospital Melbourne, Fitzroy, VIC 3065 Australia

**Keywords:** Basal radial glia, Cerebral cortex, Cortical development, Cortical folding, Outer subventricular zone, RNA-Seq

## Abstract

Gyrification of the cerebral cortex is essential for healthy brain development, and disruptions to this process lead to severe structural and functional abnormalities associated with long-term behavioural and psychiatric consequences. Basal radial glial cells (bRGC), residing in the expanded outer subventricular zone (oSVZ), are thought to drive gyrification. However, the transcriptional programs guiding this process remain poorly defined. We aimed to profile gene expression within the oSVZ of the developing ferret cortex, a species that displays postnatal gyrification, at two key stages of cortical folding, capturing its onset and progression. Discrete regions of the oSVZ were microdissected beneath the coronal gyrus and suprasylvian sulcus at the onset (P5, *n* = 5) and middle (P15, *n* = 5) of gyrification in the ferret. We performed RNA sequencing, differential expression analysis with gene ontology and pathway analyses with RT-qPCR validation of candidate genes. The results revealed spatially enriched gene sets associated with progenitor proliferation, self-renewal, and neurogenesis, as well as temporal transcriptional enrichment for axonogenesis and extracellular matrix regulation. RT-qPCR validated spatial and temporal expression differences, confirming consistent trends among some key folding-associated candidate genes. However, some genes (namely *SOX9*, *AJAP1*, and *SEMA3D*) showed inverse expression patterns compared to those detected by RNA-Seq and could not be considered validated. Together, these data define the molecular architecture of gyrification, providing a comprehensive transcriptional atlas of the oSVZ in the developing brain. Our findings advance understanding of how coordinated gene networks shape cortical folding, offering insights into the evolutional expansion of the mammalian brain.

**Supplementary Information:**

The online version contains supplementary material available at https://doi.org/10.1007/s00429-026-03178-6.

## Introduction

The mammalian cerebral cortex exhibits evolutionary diversity in size and shape, most noticeably in the formation of cortical folds. The evolution of cortical gyrification arises from tightly coordinated neurodevelopmental processes, including neural progenitor proliferation, differentiation, and neuronal migration, that collectively shape cortical architecture. Although phylogenetic evidence suggests that gyrencephaly is the ancestral mammalian condition and that lissencephaly in some lineages reflects secondary loss (Lewitus et al. 2014; Llinares-Benadero and Borrell 2019; Kelava et al. 2013), the molecular mechanisms that govern the emergence and regional patterning of cortical folds remain poorly understood. While fundamental aspects of cortical development remain conserved across mammals, species-specific differences in gene expression, the timing of neurogenesis, and biomechanical influences drive substantial variation in cortical morphology, particularly in the emergence and patterning of gyri and sulci (Pinson and Huttner 2021). The roles of founder progenitor populations, including radial glial cells (RGCs) and intermediate progenitor cells (IPCs), are largely conserved across species (Barresi et al. 2024; Del-Valle-Anton and Borrell 2022). However, comparative analyses reveal that basal radial glial cells (bRGCs), a specialised glial progenitor subtype, are essential for gyrification (Heide et al. 2020; Matsumoto et al. 2020). This process relies on the presence of an expanded outer subventricular zone (oSVZ), a proliferative compartment enriched with bRGCs that is characteristic of gyrencephalic species (de Juan Romero et al. 2015; Hansen et al. 2010; Reillo et al. 2011).

bRGCs possess district molecular and functional profiles compared with other neural progenitor cells (Pollen et al. 2015). Transcriptomic analyses in humans, ferrets, and macaques, provide compelling cellular and genetic evidence implicating bRGCs as key drivers of cortical folding (de Juan Romero et al. 2015; Florio et al. 2015, 2017; Singh et al. 2024; Del-Valle-Anton et al. 2024). Dysregulated bRGC function contributes to cortical malformations such as microcephaly (Wang et al. 2022a), and abnormal gyrification is increasingly recognised as a feature of neurodevelopmental disorders including schizophrenia, autism spectrum disorders, and epilepsies such as Rett Syndrome (Keidar et al. 2019; Sasabayashi et al. 2021).

Malformations of cortical development and folding result in alterations to neuronal circuitry and network connectivity, leading to behavioural impairments that vary in severity and presentation depending on the affected brain regions. Patients often exhibit a combination of motor, language, and social deficits, as seen in conditions such as cerebral palsy, and frequently experience early-onset neonatal seizures that persist into adulthood (Russ et al. 2025). Dysregulation of the molecular mechanisms that regulate RGC function, reviewed extensively by our group (Barresi et al. 2024), contribute to adverse neurodevelopmental and psychiatric outcomes. Despite significant advances, the molecular drivers of cortical folding remain incompletely defined. Given their role in brain development, understanding bRGC biology in both normal and pathological contexts is critical for understanding mechanism underlying cortical malformation and associated behavioural outcomes.

The development and positioning of primary gyri and sulci are highly conserved within species (Armstrong et al. 1995; Hasan et al. 2011; Lohmann et al. 1999), suggesting a strong genetic basis for cortical folding (Barresi et al. 2024; de Juan Romero and Borrell 2017; de Juan Romero et al. 2015; Singh et al. 2024; Del-Valle-Anton et al. 2024). To identify the molecular mechanisms underlying this process, researchers have analysed transcriptomes of cortical regions destined to form gyri or sulci before their emergence. This approach has revealed candidate genes and pathways that may drive fold formation, and in the ferret (de Juan Romero et al. 2015; Singh et al. 2024), has identified region-specific transcriptomic signatures associated with early stages of gyrification. However, these studies lack spatio-temporal resolution during active folding due to performing pre- versus adult post-folded cortical comparisons. Thus, building on these findings, our study addresses a major knowledge gap, and investigates gene expression with the oSVZ beneath a conserved gyrus and sulcus during the onset and progression of folding, providing both spatial and temporal insight into molecular programs driving gyrification.

The ferret is an established model for studying cortical folding because it is born lissencephalic and undergoes rapid gyrification between postnatal days (P) 4 and 6, completing folding by P28 (Sawada and Watanabe 2012; Smart and McSherry 1986a). We performed RNA sequencing (RNA-Seq) at two key neurodevelopmental stages to capture the onset and progression of cortical folding, characterising gene expression in the oSVZ of a conserved gyrus and sulcus of the parietal cortex, a region not previously examined. We additionally compared spatial and temporal transcriptional profiles to identify candidate genes, pathways, and biological processes involved in gyrification, and verified differential expression of key genes using reverse transcription polymerase chain reaction (RT-qPCR).

The oSVZ is a critical proliferative compartment in gyrencephalic species, enriched in bRGCs that contribute to cortical expansion and folding. Comparative studies have shown that oSVZ size, cell composition, and proliferative dynamics correlate with gyrencephaly, underscoring its central role in the evolutionary diversification of cortical architecture. Although bRGCs and the oSVZ are recognised as central drivers of cortical folding, the spatial and temporal transcriptional programs underlying gyrus- and sulcus-specific development remain largely unexplored. By profiling gene expression in the ferret parietal oSVZ at two critical stages of cortical folding and confirming the expression of selected candidates by RT-qPCR, our study provides new insight into the spatially restricted molecular programs that may orchestrate local differences in progenitor behaviour and drive gyral and sulcal formation on the cortex. More broadly, these data provide a transcriptional reference of newly identified enriched gene sets and pathways that advance our understanding of the pathogenesis of neurodevelopmental disorders associated with abnormal cortical folding.

## Results

### Gene expression differences at the onset of folding reflect subtle transcriptional modulation, whereas temporal transcriptional differences capture gross morphological changes of active folding

We first investigated whether regional differences in gene expression were present between P5 and P15 gyral and sulcal oSVZ. Pairwise multiple comparisons identified only nine significant differentially expressed genes (DEGs) between the coronal gyrus (CG) and suprasylvian sulcus (SSS) oSVZs at P5 (*FDR* < 0.05), namely, *FABP7*, *RPS19*, *CBLB*, *GAPDH*, *NETO*, *H2AC25*, *PIK3R1*, *PHF20L1*, and two unannotated genes, likely corresponding to *RPL32* and *RPL21* based on predicted annotations (Table 1). We observed no significant gene expression differences between the CG and SSS at P15 (*FDR* > 0.05).

**Fig. 1:**
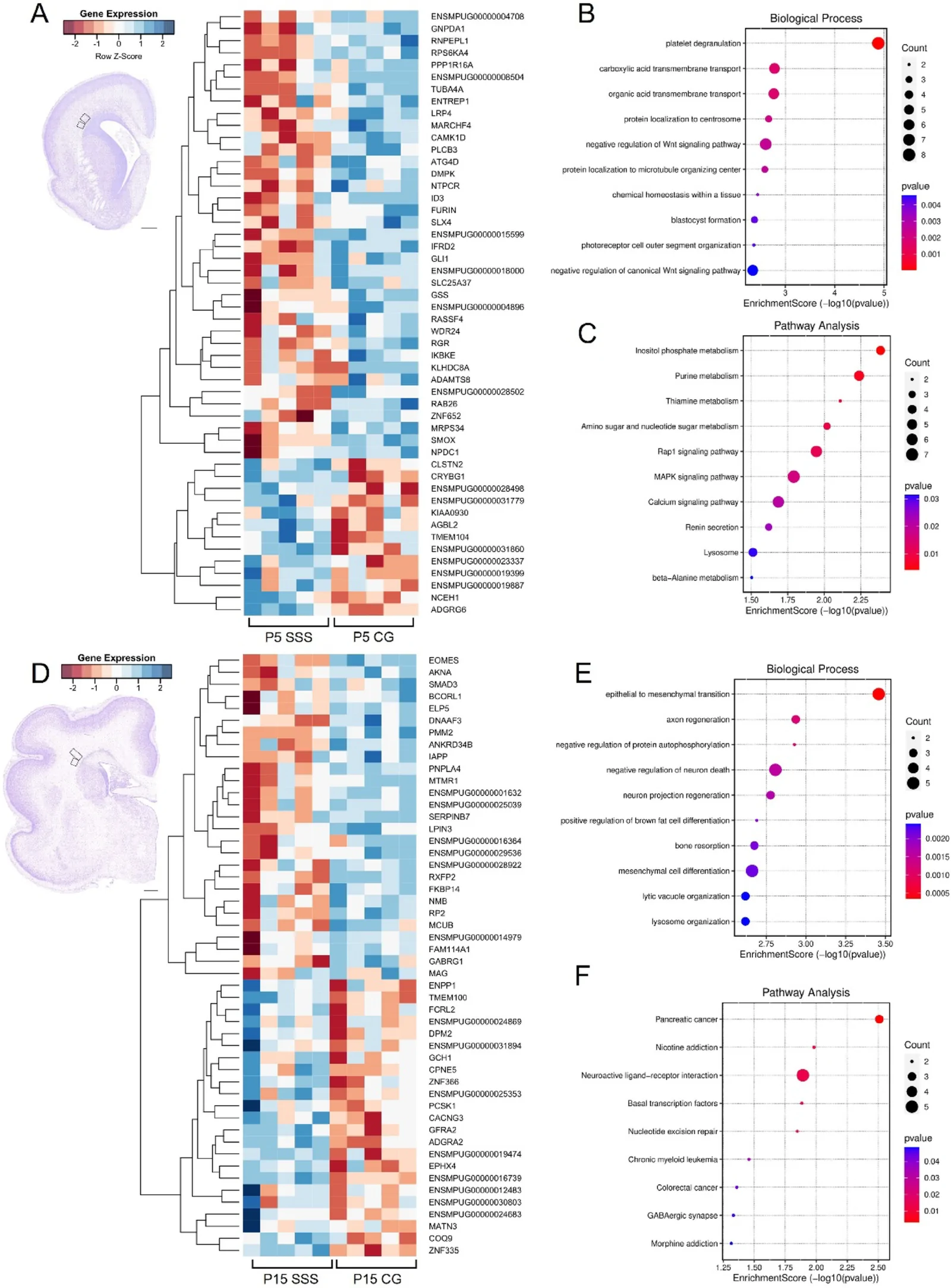
Differential Gene Expression in the oSVZ between the CG and SSS at P5 and P15 **A** Heatmap of unsupervised hierarchical clustering of differentially expressed genes (DEGs) in the outer subventricular zone (oSVZ) between the coronal gyrus (CG) and suprasylvian sulcus (SSS) at postnatal day 5 (P5; top 50 absolute logFC > 2, unadj. *p* < .05). Nissl-stained P5 coronal brain section illustrates the CG and SSS oSVZ regions analysed and the subsequent DEG list was generated from this comparison. **B** Top ten enriched biological processes between the CG and SSS at P5 derived from gene ontology (GO) analysis. **C** Top ten enriched pathways between the CG and SSS at P5 derived from KEGG pathway analysis. **D** Heatmap of unsupervised hierarchical clustering of DEGs in the oSVZ between the CG and SSS at P15 (top 50 absolute logFC > 2, unadj. *p* < .05). Nissl-stained P15 coronal brain section illustrates the CG and SSS oSVZ regions analysed and the subsequent DEG list was generated from this comparison. **E** Top ten enriched biological processes between the CG and SSS at P15 derived from GO analysis. **F** Top ten enriched pathways between the CG and SSS at P15 derived from KEGG pathway analysis. Heatmaps were generated using the heatmap.2 function from the gplots package (v3.1.3.1). Key for **A** and **D**: raw Z-scores. Key for **B**,** C**,** E**, and **F** (Count, black circles): size of circles indicative of number of genes in GO term/KEGG pathway. GO and KEGG analyses were conducted on adjusted DEGs lists comprising 217 genes (173 annotated) for the CG versus SSS comparison at P5, and 109 genes (76 annotated) at P15. For the P5 versus P15 comparisons, adjusted lists included 652 genes (570 annotated) in the CG and 704 genes (624 annotated) in the SSS. Scale Bars = 1 mm

Interestingly, several DEGs with large fold change (FC) between regions at P5 did not reach statistical significance. For example, *TUBA4A*, *RAB26*, and *SMOX* showed higher expression in the CG (FC > 7.31, *FDR* > 0.05), while *U6*, *SEMA3D*, and *GFRA2* were lower in the CG (FC > 5.66, *FDR* > 0.05). At P15, *PGAP4*, *RXFP2*, *EOMES*, and *OSTN* were among the most upregulated genes in the CG, whereas *ADGRA2* and *EPHX4* were the top upregulated genes in the SSS (Fig. 1A, D). Together, this data may reflect a subtle regional transcriptional modulation where biologically meaningful gene expression differences (reflected in large fold change differences) may not have reached statistical significance.

**Fig. 2:**
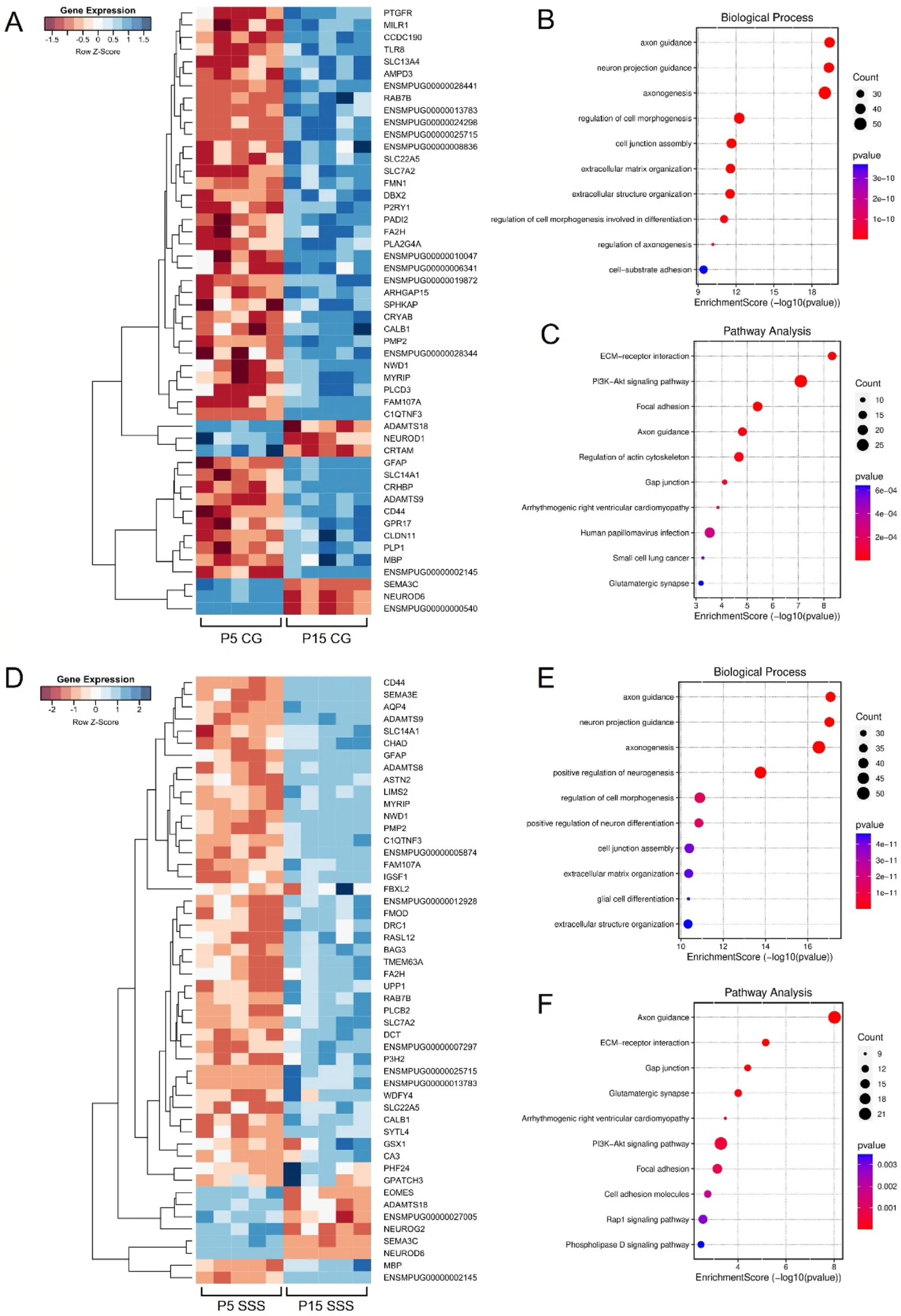
Differential gene expression in the oSVZ beneath the CG or the SSS at the onset and mid-point of cortical folding **A** Heatmap of unsupervised hierarchical clustering of differentially expressed genes (DEGs) in the outer subventricular zone (oSVZ) between the coronal gyrus (CG) at postnatal day 5 (P5) compared to P15 (top 50 absolute logFC > 2, FDR < 0.01). **B** Top ten enriched biological processes before compared to during cortical folding in the CG oSVZ derived from gene ontology (GO) analysis. **C** Top ten enriched pathways before compared to during cortical folding in the CG oSVZ derived from KEGG pathway analysis. **D** Heatmap of unsupervised hierarchical clustering of DEGs in the oSVZ between the suprasylvian sulcus (SSS) at P5 compared to P15 (top 50 absolute logFC > 2, FDR < 0.01). **E** Top ten enriched biological processes before compared to during cortical folding in the SSS oSVZ derived from GO analysis. **F** Top ten enriched pathways before compared to during cortical folding in the SSS oSVZ derived from KEGG pathway analysis. Heatmaps were generated using the heatmap.2 function from the gplots package (v3.1.3.1). Key for **A** and **D**: raw Z-scores. Key for **B**,** C**,** E**, and **F** (Count, black circles): size of circles indicative of number of genes in GO term/KEGG pathway. GO and KEGG analyses were conducted on adjusted DEGs lists comprising 217 genes (173 annotated) for the CG versus SSS comparison at P5, and 109 genes (76 annotated) at P15. For the P5 versus P15 comparisons, adjusted lists included 652 genes (570 annotated) in the CG and 704 genes (624 annotated) in the SSS

Comparison of the CG oSVZ between P5 and P15 revealed 1,593 significant DEGs, while 1,729 significant DEGs were identified in the SSS oSVZ over the same time period (*FDR* < 0.05, Table 2). *SEMA3C* emerged as the most DEG, showing a marked decrease at P15 compared to P5 – approximately 20-fold in the CG and 13-fold in the SSS. Across both regions, many key genes exhibited increased expression at P15 relative to P5 likely reflecting distinct maturational and structural changes from the onset to middle of cortical folding (Fig. 2A, D).

**Table 1:** Top ten differentially expressed genes at P5 and P15 between oSVZ regions

P5 CG oSVZ^+^ → P5 SSS oSVZ				P15 CG oSVZ^+^ → P15 SSS oSVZ			
Gene ID	logFC	*p* value	FDR adj. *p* value	Gene ID	logFC	*p* value	FDR adj. *p* value
*FABP7*	0.59	< 0.001	0.000915***	*SST*	-0.95	< 0.001	0.377
*RPS19*	0.68	< 0.001	0.002663**	*NPY*	0.78	< 0.001	0.966
*CBLB*	-0.85	< 0.001	0.003707**	*TMEM259*	-1.01	< 0.001	0.966
*GAPDH*	0.62	< 0.001	0.017363*	*SPOCK1*	0.58	< 0.001	0.966
*NETO2*	-0.52	< 0.001	0.041376*	*EOMES*	2.44	< 0.001	0.966
*H2AC25*	0.73	< 0.001	0.046258*	*FABP7*	0.37	< 0.001	0.966
*RPL32*^a^/ENSMPUG00000017189	0.48	< 0.001	0.046258*	*VIM*	0.43	< 0.01	0.966
*RPL21*^a^/ENSMPUG00000009353	0.39	< 0.001	0.047133*	*HCFC1*	-0.97	< 0.01	0.966
*PIK3R1*	-0.75	< 0.001	0.047133*	*DOP1A*	-0.53	< 0.01	0.966
*PHF20L1*	-0.55	< 0.001	0.051136	*RIMS2*	-0.42	< 0.01	0.966

*CG* coronal gyrus; *oSVZ* outer subventricular zone; *P* postnatal day; *SSS* suprasylvian sulcus

*FDR significant at < 0.05, **FDR significant at < 0.01, ***FDR significant at < 0.001

^a^Genes without annotation but have available predicted annotations sourced by inferred homology in UniProt database. Negative logFC values are indicative of lower expression in the initial condition (denoted by ‘^+^’). Conversely, positive logFC values indicative of greater expression in the initial condition

**Table 2:** Top ten differentially expressed genes for each oSVZ region when compared across developmental ages

P5 CG oSVZ^+^ → P15 CG oSVZ				P5 SSS oSVZ^+^ → P15 SSS oSVZ			
Gene ID	logFC	*p* value	FDR adj. *p* value	Gene ID	logFC	*p* value	FDR adj. *p* value
*SEMA3C*	4.318584	< 0.001	3.69e^− 15^***	*SEMA3C*	3.705823	< 0.001	1.38e^− 13^***
*NEUROD6*	3.887023	< 0.001	1.22e^− 13^***	*NEUROD6*	3.840655	< 0.001	1.50e^− 13^***
*APC*	-2.53487	< 0.001	9.00e^− 13^***	*APC*	-2.44002	< 0.001	2.40e^− 12^***
*OGN*	-3.29046	< 0.001	1.10e^− 11^***	*ANGPT1*	-3.14252	< 0.001	3.39e^− 12^***
*SPARCL1*	-2.35639	< 0.001	3.36e^− 11^***	*ANK2*^a^/ ENSMPUG00000008309	-2.02917	< 0.001	1.05e^− 11^***
*ANGPT1*	-2.70205	< 0.001	8.02e^− 11^***	*SPARCL1*	-2.41008	< 0.001	1.59e^− 11^***
ENSMPUG00000004182^b^	-2.22711	< 0.001	8.02e^− 11^***	ENSMPUG00000004182^b^	-2.21231	< 0.001	8.65e^− 11^***
*ANK2*^a^/ ENSMPUG00000008309	-1.82543	< 0.001	9.94e^− 11^***	*OGN*	-2.93182	< 0.001	8.65e^− 11^***
*DDAH2*	2.565377	< 0.001	1.56e^− 10^***	*VCAN*	-1.67311	< 0.001	3.00e^− 10^***
*GRIA4*	-2.7036	< 0.001	1.79e^− 10^***	*CD44*	-4.19292	< 0.001	3.00e^− 10^***

*CG* coronal gyrus; *oSVZ* outer subventricular zone; *P* postnatal day; *SSS* suprasylvian sulcus

***FDR significant at < 0.001

^a^Genes without annotation but have available predicted annotations sourced by inferred homology in UniProt database

^b^Genes without annotations and no available predicted annotation from other databases. Negative logFC values are indicative of lower expression in the initial condition. Conversely, positive logFC values indicative of greater expression in the initial condition (denoted by ‘^+^’)

### Identification of novel biological processes and pathways associated with spatio-temporal progression of folding in the ferret

Comparisons between CG and SSS produced only nine significant DEGs at P5 and none at P15. For completeness, we performed gene ontology (GO) and pathway analyses on the nine significant genes; however, two were unannotated, leaving only seven for analysis. With such a limited number of annotated genes, we did not obtain meaningful GO enrichment or KEGG pathway results (data not shown). Consequently, we applied boarder inclusion criteria to generate viable gene lists for further analysis.

At P5, the most enriched biological process between the CG and SSS was platelet degranulation (*FDR* < 0.05; Fig. 1B; Supplementary Table 1). Genes enriched within this process included *CYB5R1*, *CLU*, *TUBA4A*, *LYN*, *HGF*, *PDGFA*, *CTSW*, and *VEGFB*. No other biological processes, cellular components, molecular functions, or pathways achieved significance after FDR adjustment. The top enriched pathway was inositol phosphate metabolism, involving *PI4KB*, *PLCB3*, *MTMR1*, and *ALDH6A1*, although this did not remain significant following FDR adjustment (Fig. 1C; Supplementary Table 2). At P15, the most enriched biological process between the CG and SSS was epithelial to mesenchymal transition (Fig. 1E; Supplementary Table 3). Enriched genes included *EOMES*, *TMEM100*, *BCL9L*, *AKNA*, and *SMAD3*, though none reached statistical significance after FDR adjustment. The top enriched pathway was pancreatic cancer, comprising *RALGDS*, *SMAD3*, and *E2F3*, which also did not remain significant following FDR adjustment (Fig. 1F; Supplementary Table 4).

Between P5 and P15 within the CG, 377 biological process terms were significantly enriched (*FDR* < 0.05). The most enriched were axon guidance and neuron projection guidance (Fig. 2A, B; Supplementary Table 5), comprising semaphorins (*SEMA3C*,* SEMA3D*,* SEMA3E*,* SEMA5A*,* SEMA5B*,* SEMA6A*), cell adhesion molecules including laminins (*LAMA1*,* LAMB2*), transcription factors (*TBR1*,* FOXG1*), microtubule-related proteins (*TUBB3*,* TUBB2B*,* KIF5A*, and *KIF5C*), among other enriched genes.

Following FDR adjustment, 28 molecular function and 74 cellular component terms showed significant enrichment. The top molecular function was actin binding, including genes such as *EPB41L2*,* SVIL*,* SCIN*,* SPTBN1*, and *DST*. The top cellular component was collagen-containing extracellular matrix (ECM), with genes including *OGN*,* SPARCL1*,* ANGPT1*,* ADAMTS9*, and *APOE*. Thirteen pathways were significantly enriched (*FDR* < 0.05; Fig. 2C; Supplementary Table 6). The top enriched pathway was ECM-receptor interaction, comprising numerous integrin (*ITGB8*,* ITGA2*,* ITGA6*,* ITGA7*), laminin (*LAMA1*,* LAMA4*,* LAMB2*,* LAMC1*), and collagen-related genes (*COL9A2*,* COL4A4*), as well as other ECM-related genes such as *CD44*,* FRAS1*,* FN1*,* DAG1*, and *TNR*.

Between P5 and P15 in the SSS, 428 biological process terms showed significant enrichment (*FDR* < 0.05; Fig. 2E; Supplementary Table 7). The most enriched biological processes matched those identified in the CG (axon guidance and neuron projection guidance) and involved similar gene subsets, including semaphorins, laminins, transcription factors, and microtubule-related proteins. Following FDR adjustment, 20 molecular function and 71 cellular component terms were significantly enriched. The top molecular function was glycosaminoglycan binding, including multiple ECM-associated genes such as *VCAN*,* CD44*, and *HAPLN1*. The top cellular component was collagen-containing ECM, comprising many similar ECM-associated genes such as *VCAN*,* HAPLN1*,* SPARCL1*,* OGN*,* DAG1*, and a subset of laminins. Seven pathways were significantly enriched following FDR adjustment (Fig. 2F; Supplementary Table 8), including ECM-receptor interaction and PI3K-AKT signalling, both containing many of the aforementioned enriched genes.

In addition to top DEG lists generated by traditional analyses, we performed a thorough literature search to cross-reference salient genes with known enrichment in bRGCs during gyrification or their association with cortical folding malformations. Selection of candidate genes was guided by two complementary criteria: (i) large fold-change magnitude from RNA-Seq results, and (ii) peer-reviewed evidence from literature search implicating candidate genes in bRGC proliferation, progenitor self-renewal, cortical folding, or associated neurodevelopmental malformations. From this, a small subset of candidates were chosen for RT-qPCR validation: (1) *FOXM1*, (2) *CDK6*, (3) *SOX9*, (4) *FGFR2*, (5) *LIFR*, (6) *STAT3*, (7) *LGALS3BP*, (8) *GLI1*, and (9) *PRUNE1*. Their pattern of expression and proposed role in gyrification are summarised in Table 3.

**Table 3:** Differential expression profile and proposed roles of validation targets in gyrification

Target	Differential expression profile (RNA-Seq)	Biological function and hypothesised role in gyrification
*SOX9*	1.40x ↑ expression in the CG oSVZ vs. SSS at the onset of cortical folding 1.88–2.58x ↑ expression at P15 compared to P5 in both ROIs	Required for proliferation and high enrichment in gyrencephalic germinal zones^a^. Hypothesised differential expression between gyri and sulci promotes greater proliferation and expansion in gyri
*FOXM1*	1.82x ↑ expression in the CG oSVZ vs. SSS at the onset of cortical folding	Required for proliferation and implicated in gyrification phenotype in mice^b^. Hypothesised greater expression in gyri oSVZ promotes greater proliferation and expansion than in sulci
*pSTAT3*	*STAT3* approximately equal between CG and SSS oSVZ at P5 and P15 2.55–2.77x ↑ expression at P15 compared to P5 in both ROIs	pSTAT3 promotes bRGC proliferation and self-renewal^c^. Given that *STAT3* expression is approximately equal between ROIs, the post-translational phosphorylation could differentially regulate this gene between gyri and sulci
*FOXC1*	3.56x ↑ expression in the CG oSVZ vs. SSS at the onset of cortical folding 3.72x ↑ expression from P5 to P15 in the SSS oSVZ 1.37x ↑ expression in the CG oSVZ at P15 compared to P5	Required for cell proliferation^d^. Hypothesised differential expression between gyri and sulci promotes greater proliferation and expansion in gyri. Based on RNA-Seq data, differential expression may be more important at the onset of folding, equalising between ROIs by the middle of folding
*GLI1*	6.50x ↑ expression in the CG oSVZ vs. SSS at the onset of cortical folding Not detected at P15	Required for cell proliferation and differential expression between gyri and sulci corroborated by previous research^e^. Hypothesised differential expression between gyri and sulci promotes greater proliferation and expansion in gyri via SHH signalling cascade
*PI4KB*	2.62x ↑ expression in the SSS oSVZ vs. CG at the onset of cortical folding.	A novel candidate with limited ontology information available, however could be involved in cellular signalling and trafficking within the Golgi network^f^ and may be involved with other genes and proteins to promote cortical folding
*TUBA4A*	13.64x ↑ expression in the CG oSVZ vs. SSS at the onset of cortical folding 2.07x ↑ expression in the CG oSVZ vs. SSS at the middle of cortical folding 5.28x ↑ expression in the SSS oSVZ at P15 compared to P5	Largest FC difference between the CG and SSS oSVZ at the onset of folding from the RNA-Seq data. While no link to gyrification has been established, *TUBA4A* was selected as a target for exploratory follow up of the large FC detected in the RNA-Seq
*AJAP1*	4.03x ↑ expression in the SSS oSVZ vs. CG at the onset of cortical folding.	Hypothesised to promote sulcal invagination and restrict cortical growth in sulcal regions by reducing neuronal migration and increasing tissue adhesion in the ECM^g^
*PRUNE1*	3.86x ↑ expression in the CG oSVZ vs. SSS at the onset of cortical folding 1.50x ↑ expression in the CG oSVZ vs. SSS at the middle of cortical folding	Mutations in *PRUNE1* implicated in microcephaly^h^. The current study is the first to identify *PRUNE1* as a differentially expressed gene candidate between gyri and sulci. Hypothesised to promote greater proliferation and expansion in gyri than sulci
*SEMA3D*	6.23x ↑ expression in the SSS oSVZ vs. CG at the onset of cortical folding 10.22x ↑ expression in the CG oSVZ at P15 compared to P5 2.13x ↑ expression the SSS oSVZ at P15 compared to P5	Involved in axonal guidance^i^. Hypothesised to send attractive cues beneath sulci to promote axonal innervation into the gyri

*CG* coronal gyrus; *ECM* extracellular matrix; *oSVZ* outer subventricular zone; *P* postnatal day; *RNA-Seq* RNA sequencing; *SHH* sonic hedgehog; *SSS* suprasylvian sulcus

^a^Fietz et al. (2012), Güven et al. (2020), ^b^Wang et al. (2022b), ^c^Pollen et al. (2015), ^d^Zarbalis et al. (2007), ^e^de Juan Romero et al. (2015), ^f^Zhao et al. (2024), ^g^Di et al. (2018), ^h^Zollo et al. (2017), ^i^Wolman et al. (2004)

At P5, *STAT3*,* LIFR*,* FGFR2*,* CDK6*, and *LGALS3BP* showed similar expression levels in the oSVZ beneath both the CG and SSS. However, their expression was higher at P5, coinciding with the onset of cortical folding, than at P15 in both regions (Table 3). In contrast, *FOXM1*,* SOX9*,* GLI1*, and *PRUNE1* showed higher expression in the CG compared to the SSS at P5 but equalised between regions by P15. Of these, only *GLI1* met the inclusion criteria for GO and KEGG pathway analysis in the CG versus SSS comparison at P5. *GLI1* was associated with several enriched biological processes and pathways, including multiple Wnt signalling regulatory pathways, regulation of neural precursor cell proliferation, positive regulation of cell cycle phase transition, and regulation of DNA replication. KEGG pathway analysis also revealed *GLI1* enrichment in the Hedgehog signalling pathway. By P15, *STAT3* and *LIFR* expression increased in both CG and SSS oSVZs, while *SOX9* and *PRUNE1* expression increased only in the SSS. The remaining candidate genes maintained relatively stable expression across time within each region.

### RT-qPCR confirmed expression of only a subset of selected candidate genes

**Fig. 3:**
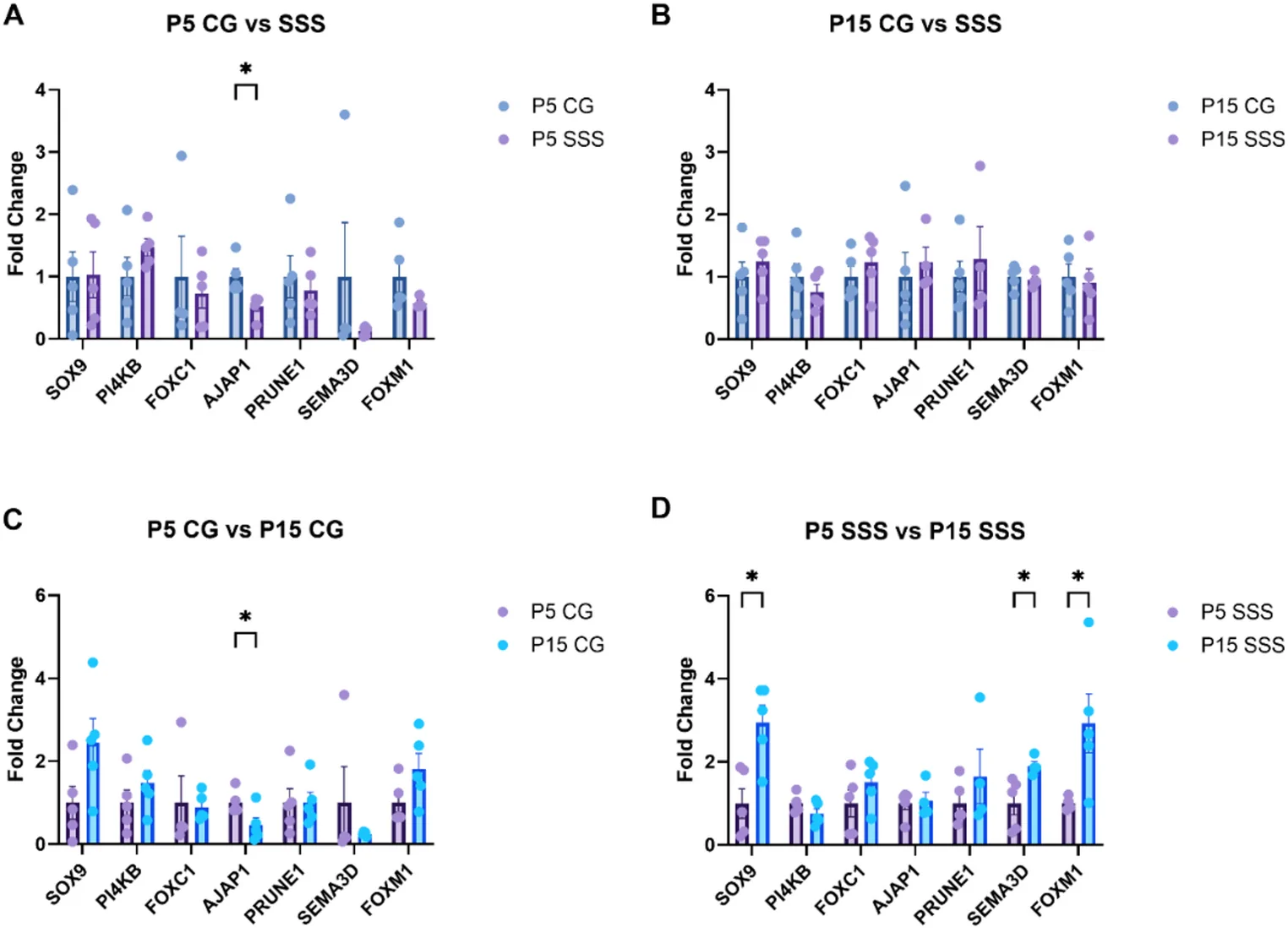
Independent Sample t-tests of RT-qPCR Data from P5 and P15 ferret coronal Gyrus and suprasylvian sulcus (**A**, **B**). Independent sample t-tests for each target between the coronal gyrus (CG) and suprasylvian sulcus (SSS) outer subventricular zones (oSVZs) at the onset (postnatal day 5, P5) and middle (P15) of cortical folding. *AJAP1* was significantly increased in the CG at P5, and no targets reached statistical significance at P15. **C**, **D** Time-wise independent sample t-tests in each region from the onset to the middle of cortical folding found that *AJAP1* significantly decreased in the CG, while *SOX9*, *SEMA3D*, and *FOXM1* significantly increased in the SSS, throughout folding. Data presented as mean and SEM. *p < .05. *RT-qPCR* quantitative real time polymerase chain reaction

As an additional validation, we performed RT-qPCR on the subset of pre-selected gene candidates (Table 3). *GLI1 and TUBA4A* were subsequently excluded from RT-qPCR analysis due to undetermined Ct values. At P5, only *AJAP1* showed significantly lower expression in the SSS compared to the CG (Relative FC = 0.53, *t*(8) = 3.17, *p* = .01); no other genes differed significantly between regions (Fig. 3A). Consistent with the RNA-Seq findings, we observed no significant differences between the CG and SSS at P15 (Supplementary Table 9; Fig. 3B). However, discrepancies appeared at P5 for *SOX9*,* AJAP1*, and *SEMA3D*, which showed expression patterns opposite to those predicted by RNA-Seq. In contrast, *PI4KB*,* FOXC1*,* PRUNE 1*, and *FOXM1* displayed expression patterns consistent with the RNA-Seq data, although with reduced fold change magnitude.

In the CG at P15, *AJAP1* expression decreased significantly compared to P5 (Relative FC = 0.46, *t*(8) = 2.47, *p* = .04), a finding that contradicted RNA-Seq results. In contrast, expression of *SOX9* (Relative FC = 2.95, *t*(8) =-3.56, *p* = .01), *SEMA3D* (Relative FC = 1.90, *t*(7) =-3.08, *p* = .03), and *FOXM1* (Relative FC = 2.93, *t*(7) =-2.40, *p* < .05) increased significantly at P15 compared to P5 in the SSS, consistent with RNA-Seq findings (Supplementary Table 10; Fig. 3C, D). Taken together, the RT-qPCR confirmed consistent expression patterns in *PI4KB*,* FOXC1*,* PRUNE1*, and *FOXM1*, however was unable to confirm expression of *GLI1*,* TUBA4A*,* SOX9*,* AJAP1*, or *SEMA3D* in these samples.

## Discussion

Differential gene expression analysis identified transcriptional programs, biological processes, and pathways enriched between the developing CG and SSS at the onset and midpoint of cortical folding in the ferret. The most pronounced transcriptional changes occurred within each region over time, between P5 and P15 in both the CG and SSS oSVZs, reflecting the developmental transition from active neurogenesis to gliogenesis and structural maturation of the cerebral cortex. In contrast, regional differences between the CG and SSS at the onset of cortical folding were more subtle and may represent early molecular priming events that establish local distinctions in progenitor behaviours and ultimately influence fold formation.

### Differential gene expression may prime the cortex for cortical folding

The most enriched pathway differentiating the developing CG and SSS at P5 was platelet degranulation, encompassing genes linked to progenitor cell proliferation and self-renewal, including *CYB5R1*, *CLU*, *TUBA4A*, *LYN*, *HGF*, *PDGFA*, *CTSW*, and *VEGFB*. Among these, *TUBA4A* showed the greatest fold change, 13.64-fold more highly expressed in the CG. Mutations in *TUBA4A* have been implicated in amyotrophic lateral sclerosis (Smith et al. 2014), a condition in which cortical folding may be altered (Zhang et al. 2017). Although a direct link between *TUBA4A* and gyrification has not been established, other tubulin-related genes, such as *TUBA1A* and *TUBB3*, are associated with cortical malformations (Romaniello et al. 2014). Given the pronounced expression of *TUBA4A* in the CG oSVZ at P5, its established role in microtubule assembly, and the central importance of microtubules in cell division and cortical organisation, *TUBA4A* may contribute to basal progenitor proliferation or play a pivotal role in cortical folding. Consequently, mutations in *TUBA4A* could plausibly result in folding abnormalities analogous to other tubulinopathies, a hypothesis that warrants further investigation. Additionally, although platelet degranulation has been extensively studied in the context of ischemic stroke (McCabe et al. 2004; Liu et al. 2022), thrombosis (van der Meijden and Heemskerk 2019), and tumour metastasis (Tesfamariam 2016), its potential involvement, and that of its genetic regulators, in neurodevelopment and cortical morphogenesis remains largely unexplored.

Recent evidence suggests that platelets may exert more dynamic roles in brain development and function than previously thought (Burnouf and Walker 2022). For example, platelets can promote neurogenesis via the SVZ vasculature in vitro (Hayon et al. 2012), as well as in the adult rat SVZ (Kazanis et al. 2015) and hippocampal dentate gyrus (Leiter et al. 2019). The enrichment of this pathway during early gyrification therefore raises the intriguing possibility that platelet-associated mechanisms may contribute to oSVZ progenitor expansion, proliferation, or other aspects of gyrification. Alternatively, genes classified within this pathway may serve dual roles, functioning in both platelet biology and brain development.

We also identified several growth factors, including *VEGF2*, *PDGFA*, and *HGF*, enriched in the oSVZ beneath the CG compared to the SSS at P5. Notably, *PDGF* and *VEGF2* have been shown to act synergistically with *FGF2*, a factor previously implicated in gyrification, to enhance sphere size and activate phosphorylated ERK/AKT signalling in 3D mouse neural spheres enriched with platelet microparticles (Hayon et al. 2012). Given that FGFs and ERK/AKT signalling are well-established mediators of bRGC genesis, proliferation, and cortical folding (Li et al. 2017; Ju et al. 2016), the concurrent enrichment of growth factors such as *PDGFA* and *VEGF2* in the present study suggests that a platelet-associated signalling pathway may be essential in driving gyrification.

Several growth factors identified in the present study, including *VEGF*, have been implicated in promoting neurogenesis, guiding neuronal migration, and directing axonal guidance, as well as acting as chemoattractants for undifferentiated, migrating neurons in FGF-activated neural progenitor cells (Zhang et al. 2003; Mackenzie and Ruhrberg 2012; Rosenstein et al. 2010). However, conflicting evidence suggests that VEGF receptors may be absent in neural progenitors (Javaherian and Kriegstein 2009), raising questions about the precise role of *VEGF* in cortical development. Resolving these discrepancies will require detailed investigations into the spatio-temporal expression of *VEGF* and its receptor during corticogenesis. Similarly, *HGF* has been linked to SVZ progenitor proliferation and self-renewal via ERK-dependent signalling, with evidence indicating that endogenous HGF activity alone is sufficient to drive mitogenesis in SVZ neural progenitors (Nicoleau et al. 2009). This aligns with our observation of increased *HGF* expression in regions exhibiting greater cortical expansion.

Several additional genes with high fold changes were upregulated in the CG oSVZ at P5, including *RAB26*, a known negative regulator of neuronal migration (Liu et al. 2021), and *SMOX*, which negatively influences neuronal differentiation (Wang et al. 2020). Conversely, genes upregulated in the SSS oSVZ at P5 included *U6*, a small nuclear RNA (Didychuk et al. 2018); *SEMA3D*, involved in axonal guidance (Wolman et al. 2004); and *GFRA2*, a proposed regulator of neuronal differentiation (Li et al. 2019). Notably, *PI4KB*, a gene enriched within the platelet degranulation pathway, was 2.62-fold more highly expressed in the SSS, although its role in brain development remains to be characterised.

Although many of these genes have not been directly studied in the context of early neurodevelopment or gyrification, they may contribute to regulating the balance between progenitor proliferation and neurogenesis, as well as axonal organisation and innervation. Dysregulation of these genes could plausibly result in premature neurogenesis, aberrant neuronal migration, or defects in axonal pathfinding, ultimately disrupting cortical folding and the structural organisation of the cerebral cortex.

To further contextualise our findings, several candidate genes previously implicated in cortical folding –*SOX9* (Güven et al. 2020), *STAT3* (Pollen et al. 2015), *FOXM1* (Wierstra and Alves 2007), *FOXC1* (Zarbalis et al. 2007), *GLI1* (Matsumoto et al. 2020), *AJAP1* (Di et al. 2018), and *SEMA3D* (Wolman et al. 2004)– were cross-referenced against our dataset to examine their expression patterns in the CG and SSS oSVZ throughout cortical folding. *FOXM1* and *GLI1* were particularly noteworthy, given their established roles in promoting cortical folding, as demonstrated in gene transfection studies (Wang et al. 2022b; Matsumoto et al. 2020), and were enriched in the CG oSVZ at the onset of gyrification. Similarly, *PRUNE1*, also enriched in the CG oSVZ, has been associated with microcephaly and autism spectrum disorder, and is thought to regulate microtubule polymerisation, a critical process for cell division and proliferation (Zollo et al. 2017). These observations highlight a subset of genes whose spatiotemporal expression patterns in the CG oSVZ align with key processes underpinning cortical folding.

RT-qPCR validation produced somewhat unexpected results, particularly regarding the direction and magnitude of mRNA expression differences. For example, *AJAP1* and *SEMA3D* exhibited inverse expression patterns between the CG and SSS at P5 relative to the RNA-Seq findings and their reported biological functions. *AJAP1* is known to enhance cell adhesion and reduce migration via the ECM (Di et al. 2018), whereas *SEMA3D* modulates axonal guidance, exerting either attractive or repulsive effects depending on context (Wolman et al. 2004). The observed upregulation of both genes in the CG compared to the SSS at P5 suggests a microenvironment characterised by increased tissue stiffness, reduced cellular migration, and potentially enhanced axonal repulsion. This transcriptional profile contrasts with the gene expression patterns typically associated with increased proliferation, neuronal migration, and tissue expansion in pre-gyral regions, processes considered prerequisite for cortical folding. Nonetheless, the expression patterns of *PI4KB*, *FOXC1*, *PRUNE1*, and *FOXM1* were generally consistent with the RNA-Seq data at P5, although the fold changes detected by RT-qPCR were substantially lower than those observed in the transcriptomic analysis. We were unable to validate *SOX9*,* AJAP1*, or *SEMA3D* in this study, and these candidates should undergo future orthogonal validation.

Technical factors may account for discrepancies between the RNA-Seq and RT-qPCR data, including differences in library preparation protocols, reverse transcription efficiency, or probe and primer specificity. Notably, the primers used for RT-qPCR were designed based on the currently available ferret genome, which remains only partially annotated. Consequently, probe and primer design may not fully capture uncharacterised transcript isoforms or may inadvertently detect transcripts absent from the annotated genome. These limitations highlight the importance of continued validation of candidate genes using complementary in vivo and in vitro approaches.

### Enrichment of ECM genes and axonogenesis in the oSVZ are critical transcriptional signatures during gyrification

By P15, the enrichment of ECM-related genes, together with axonal guidance molecules, become the dominant transcriptional signature. In both regions, DEGs were enriched in shared biological processes and pathways fundamental to neocortical expansion and maturation (Gilardi and Kalebic 2021; Smart and McSherry 1986b; Shinmyo et al. 2022), including axonogenesis, neuronal differentiation, cell morphogenesis, astrogenesis, and gliogenesis. This shift likely reflects the increasing structural complexity of the cortex and the maturation of neural circuity. Multiple collagens, laminins, and integrins were enriched in both the CG and SSS oSVZ at P15 compared to P5. Laminins and integrins provide key signalling cues to support progenitor cell maintenance and expansion (Fietz et al. 2010; Güven et al. 2020), while collagens contribute to tissue mechanics by preserving stiffness and structural integrity (Long et al. 2018). Together, these ECM molecules facilitate neuronal migration and establish the structural support necessary for both radial and tangential expansion during development and maturation.

DEGs were enriched in pathways associated with ECM-receptor interactions, ECM structure and organisation, PI3K-Akt signalling, focal adhesion, and regulation of the actin cytoskeleton, with integrins also prominently represented in cell adhesion pathways. Previous studies have reported strong enrichment of ECM components and related signalling molecules in the oSVZ compared with the VZ and inner SVZ of 14-16-week human fetal brains (approximately equivalent to P3-4 in the ferret) prior to the onset of cortical folding (Fietz et al. 2012). In our study, ECM-related gene expression was detectable in the oSVZ at P5 but was significantly upregulated by P15, indicating that ECM-associated transcriptional activity intensifies during mid-gyrification. Notably, this upregulation was comparable between the CG and SSS, suggesting that ECM-mediated signalling represents a common feature of maturing germinal zones rather than a region-specific mechanism.

The data suggests that ECM components are not only enriched at the onset of cortical folding but continue to accumulate as folding progresses. These results reinforce and align with the idea that ECM dynamics are particularly important for gyrencephalic cortical development and cerebral expansion.

### Limitations and future directions

Using a non-model organism such as the ferret introduces challenges due to its comparatively limited genome annotation relative to well-characterised species like humans or mice. This limitation reduces the accuracy of read mapping and gene summarisation in our analysis pipeline. Additionally, isolating discrete brain layers for oSVZ collection required extra experimental steps, which can impact RNA quality through increased freeze-thaw cycles and further complicates gene mapping.

Given the limited ferret annotation, we relied on a human database for GO and KEGG pathway analyses. Many DEGs exhibited overlapping roles in developmental processes, and the scarcity of data specific to gyrification made interpreting enriched pathways challenging. Consequently, our hypotheses were largely informed by existing literature, highlighting the need for future studies to validate these gene expression findings at both RNA and protein levels. These limitations underscore the importance of interpreting our transcriptional atlas as a foundational resource that can guide more targeted mechanistic studies.

We also acknowledge that microdissection of the oSVZ beneath the CG and SSS captures a spatially discrete sample at a single rostro-caudal level. Although both regions were sampled as immediately adjacent structures within the same coronal plane, the transcriptional profiles we report may reflect the regional context of this specific location, and their generalisability to gyral and sulcal oSVZ across the broader cortical mantle remains to be established in future studies.

## Conclusions

This study established a spatiotemporal transcriptional atlas of the oSVZ in the ferret brain across cortical gyrification. Using an exploratory bioinformatic approach, we identified novel genes potentially involved in cortical patterning, morphogenic growth, and neuronal and axonal guidance during early brain development. Because these genes paly an essential role in brain formation, disruptions in their expression may underlie the pathogenesis of neurodevelopmental disorders. Many identified genes exhibited overlapping roles in progenitor proliferation and self-renewal, suggesting coordinated gene regulatory mechanisms driving cortical development. Notably, gene enrichment at the onset of cortical folding in the CG supports their involvement in gyral expansion by maintaining the progenitor pool required for upper-layer neuron production, consistent with the established importance of bRGC proliferation in cortical expansion and folding. Furthermore, enrichment of ECM-related genes at P15 suggests a conserved role for the ECM in supporting growth, structural organisation, and cellular differentiation. While not predictive of gyral versus sulcal trajectories, the ECM likely provides essential scaffolding and signalling functions throughout development, potentially regulating astrogenesis and axonal guidance in a region-independent manner. Collectively, the transcriptional data generated by this study provide a valuable resource for understanding evolutionary biology, neurodevelopment, and the genetic aetiology of brain malformations.

## Materials and methods

### Animals, postmortem tissue collection, and processing

Ferret kits were obtained from a private supplier (Whittlesea, VIC), at neurodevelopmental stages corresponding to the onset (P5, *n* = 5; 3 female, and 2 male) and middle (P15, *n* = 5; 4 female, and 1 male) of cortical folding (Gilardi and Kalebic 2021; Smart and McSherry 1986a). Postnatal age was determined by the supplier, with P1 designated as the day of birth. Sex was not included as a variable due to the low sample size and uneven spread. Kits were euthanised via inhaled isoflurane followed by severing of the carotid artery. Brains were extracted, hemisected, and cut into 5 mm-thick rostrocaudal blocks (e.g., R1, R2, R3), depending on brain size (Supplementary Fig. 1). Blocks of the left cerebral hemisphere (fresh and unfixed) were placed in optimal cutting temperature (OCT, TissueTek, CA, USA) compound, snap-frozen in liquid nitrogen-cooled isopentane immediately after extraction (without cryoprotection) to preserve RNA integrity and stored at -20 °C until use.

### Tissue microdissection

Fresh-frozen, OCT-embedded tissue from the R2 region of P5 and P15 ferret brains (Supplementary Fig. 1) was sectioned at 20 μm using a cryostat (Leica Biosystems, CM1950) and mounted onto nuclease-free polyethylene naphthalate (PEN) membrane slides (ZEISS, Oberkochen, Germany). This region contained most of the CG and SSS, along with their respective subventricular regions (Supplementary Fig. 2).

Sections were air-dried for two minutes at room temperature, submerged in 95% RNase-free ethanol (ETOH) for 30 s, and Nissl-stained using the ARCTURUS™ HistoGene™ laser capture microdissection (LCM) Frozen Section Staining kit (ThermoFisher, MA, USA) following the manufacturer’s recommendations. Briefly, sections were air-dried for five minutes, then sequentially immersed for 30 s each in 75% RNase-free ETOH, RNase-free water, Nissl stain, RNase-free water, 75% RNase-free ETOH, 95% RNase-free ETOH, and finally 100% RNase-free ETOH. Sections were again air-dried at room temperature for five minutes and stored at -80 °C with desiccant beads to maintain dehydrated until LCM.

oSVZ regions were laser-captured from PEN slides into lysis buffer using the PALM MicroBeam system (Microlaser Technologies) and PALMRobo software (v3.2). For each specimen at P5 or P15, samples were generated by pooling the oSVZ extracts of either the CG or SSS across 50 consecutive coronal sections, resulting in a total of 20 samples. Samples were stored at -80 °C with desiccant beads until RNA purification and extraction.

### RNA purification and sequencing

RNA purification was performed using the RNeasy Micro Kit (QIAGEN, Australia) with an on-column DNaseI treatment, following the manufacturer’s protocol. RNA integrity and purity were assessed using an Agilent Bioanalyzer 2100 (Agilent Technologies, Australia). Approximately 2ng of RNA was used for cDNA synthesis and SPIA amplification using the Tecan RNA-Seq V2 kit. SPIA-amplified cDNA was then used for library generation using the Tecan Celero EZ DNA-Seq Library kit, with approximately nine amplification cycles (or 11 cycles for samples with < 100ng). Paired-end SPIA sequencing was performed using an Illumina NextSeq2000 sequencer (Illumina Inc, Australia) at the Monash Health Translation Precinct Medical Genomics Facility (VIC, Australia).

### Mapping and differential expression analysis

The fully annotated ferret genome (Genome: MusPutFur1.0, Toplevel unmasked; Annotations: MusPutFur1.0.108) was obtained from the Ensembl database in December 2022. Raw data were pre-processed using Trimmomatic (v0.38) (Bolger et al. 2014) with standard paired-reads settings to remove residual Illumina adapters and to set length between 36 and 100 bases. QC was performed on both untrimmed raw data and trimmed data using FastQC (v0.11.9) (Andrews 2010). Trimmed sequencing data were mapped and aligned to the reference genome using STAR (v2.7.9a) (Dobin et al. 2013) and summarised in Rstudio (v2023.06.1, Build 524) using the featureCounts function from the Rsubread package (v2.16.0) (Liao et al. 2019).

Differential expression analysis was performed using the edgeR package (v3.42.4) (Robinson et al. 2010) within the R environment (v4.3.1) via Rstudio. Raw counts were imported and filtered by age (P5 and P15) and region of interest (CG and SSS) to generate the initial DEG list. Low expression counts were removed, reducing the total from 32,057 to 11,143. Data were normalised to account for sequencing depth and library size, and the average common dispersion was calculated across groups. Differential expression was assessed using the quasi-likelihood F-Test with a Benjamin-Hochberg false discovery rate (FDR) adjustment (Lun et al. 2016), modelled for paired samples.

### Gene ontology and KEGG pathway analysis

GO enrichment and pathway analyses were performed using an online RNA-Seq data analysis and visualisation platform (http://bioinformatics.com.cn/srplot, correct October 2023). Due to limited annotation of the ferret genome, analyses were performed using human genome homologues to infer biological function.

Genes selected for GO and KEGG analyses met the following criteria: (a) for within-age comparisons, an unadjusted *p* < .05 and an absolute FC > 2 between conditions; (b) for across-age comparisons, an adjusted *p* value (i.e. *FDR*) <0.01 and absolute FC > 2 between conditions. GO terms were obtained using EBI search (Madeira et al. 2022).

### RT-qPCR

Custom TaqMan assays (ThermoFisher, MA, USA) were designed for each target gene using NCBI sequence data, spanning exons where possible. *UBE2D2* was selected as the reference (housekeeping) gene due to its logCPM expression being more comparable to the target genes, thereby reducing the risk of highly abundant housekeeping gene expression masking the detection of lower-expressing targets (Rydbirk et al. 2016).

RT-qPCR was performed on the same RNA used for RNA-Seq. cDNA synthesis was performed using the SuperScript IV VILO Master Mix kit (Applied Biosystems, MA, USA) following the manufacturer’s instructions. A preamplification step was included using TaqMan PreAmp Master Mix kit (Applied Biosystems, MA, US) to non-biasedly amplify target genes from low-yield RNA samples. Preamplified cDNA was diluted 1:10 in Tris EDTA buffer prior to qPCR.

qPCR was performed on the QuantStudio 7 Flex system (ThermoFisher, MA, US) using duplexed assays (Table 4), with each sample run in triplicate alongside corresponding RT-negative controls. Gene expression levels were inspected using Design and Analysis and Relative Quantification software on ThermoFisher Data Connect Online Cloud Platform. Data were exported to Microsoft Excel (v.2503, Build 16.0.18623.20116; Microsoft Corporation, WA, USA) and Ct values < 8 or > 35 were excluded as outliers. Triplicates were retained only if they fell within ±1Ct value of each other. Expression was normalised to *UBE2D2* using the ΔΔCt method to calculate fold change ratio. A fold change near 1.00 indicated no change relative to the reference condition. Independent samples t-tests were used to assess statistically significant differences in fold change using normalised ΔΔCt values, with significance set at *p* < .05.

**Table 4:** Custom designed Taqman assay ID and primer and probe pairs for RT-qPCR

Assay ID	Gene ID	Forward primer	Reverse primer	Probe
APAAKMC	*UBE2D2* ^*a*^	CTCCCACGATGGCTCTGAA	GTCCCGTGCCAGGTCATT	AATTCCTTGTGGATTCTC
APGZRGX	*FOXM1* ^*a*^	CCACAATCGCCTGAGCACTT	GGAGTTCAGGATTGGGTCGTTT	CTGCTGCTGTGATTCC
APEP3C3	*GLI1* ^*b*^	CCAGCCAGAGAGACCAACAG	CCCGCTTCTTGGTCAACTTGA	CTGAGGCCCCACTCTT
APNK2TM	*SOX9* ^*b*^	CTCCTCCTCCGGCATGAG	GCTGCACGTCGGTTTTGG	CACTCCGGGCAATCC
APMF77P	*PI4KB* ^*c*^	CCACACCCAGGCTGTTGT	GGTGGTGTCAAAGTTTTCACATTCA	CCAAGGACAAGGCTCCCT
APKCEMT	*TUBA4A* ^*c*^	CCGCATCGGTACACCTAACC	CCCACATGGACTGAGATGCA	TCGCGCTGTCGCACTT
APCFE69	*FOXC1* ^*d*^	GGAGAGATGGCGATTTGATTACAGA	CGGCAGATAGGAGGAAACTGTATTA	CAGGGCCTTTCAGTTTGT
APDJ9R6	*AJAP1* ^*d*^	AGCCGAGTACAAATCCACGTTTA	CAGGAATAAGGTGCCGATCAGA	ACGGGAACCGACCCTC
APFVWWZ	*PRUNE1* ^*e*^	GGCTATTTTCTGTCCCCATGCA	GAATGGGAGCGTTCCAGAACT	CCGCAGATCGTCATTC
AP9HU9K	*SEMA3D* ^*e*^	TCCTGTGGTCTATGGAGTCTTTACC	CACACAAACAGCTGAGCCTTT	AACCAGCTCCATCTTC

^a, b,c, d,e^Pairs denote duplex assays combinations for qPCR

## Supplementary Information

Below is the link to the electronic supplementary material.

Supplementary Material 1

Supplementary Material 2

## Data Availability

Sequencing data from this study have been submitted to the GEO database under the accession number ‘GSE304435’. All data supporting the findings of this study are available in this article and its supplementary materials. Further enquiries can be directed to the corresponding author.
