# Supplementary material for: A transcriptional atlas of gyrification reveals dynamic spatio-temporal gene expression in the developing ferret cortex
Source: Brain Struct Funct. 2026 Aug 12;231(7):118. doi: 10.1007/s00429-026-03178-6 (PMC13469505; doi:10.1007/s00429-026-03178-6)
Supplement: Supplementary file 1 [file 429_2026_3178_MOESM1_ESM.pdf]

Category	Uniquely mapped	Mapped to multiple loci	Mapped to too many loci	Unmapped: too short	Unmapped: other	Total Reads	Uniquely Mapped Reads (%)	Multi-mapped Reads (%)	Unmapped Reads (%)	Multimappe	Unmapped rea	Column1	Average Unique Mapped reads	average multimapped reads	average unmapped reads
1-19F38P5_Gyrus_OSVZ_S1_out	12697694	531938	12675	20256932	0	3.3E+07	37.90%	1.63%	60.47%	544613	20256932	Average	35.60%	1.44%	62.96%
1-19F38P5_Sulcus_OSVZ_S2_out	13235466	574964	16850	19009049	0	3.3E+07	40.31%	1.80%	57.89%	591814	19009049	SD	3.60%	0.24%	3.65%
10-19F21P15_Gyrus_OSVZ_S37_out	11376761	391251	6666	21752974	0	3.4E+07	33.93%	1.19%	64.88%	397917	21752974				
10-19F21P15_Sulcus_OSVZ_S38_out	11705303	391974	8425	22627467	0	3.5E+07	33.70%	1.15%	65.15%	400399	22627467				
2-19F39P5_Gyrus_OSVZ_S5_out	12018678	650850	11988	24030485	0	3.7E+07	32.74%	1.81%	65.46%	662838	24030485				
2-19F39P5_Sulcus_OSVZ_S6_out	11922158	636672	14542	24367128	0	3.7E+07	32.27%	1.76%	65.96%	651214	24367128				
3-19F40P5_Gyrus_OSVZ_S9_out	11854697	511154	13004	21088391	0	3.3E+07	35.42%	1.57%	63.01%	524158	21088391				
3-19F40P5_Sulcus_OSVZ_S10_out	15332775	648739	11111	25297624	0	4.1E+07	37.13%	1.60%	61.27%	659850	25297624				
4-19F42P5_Gyrus_OSVZ_S13_out	15731563	623545	13840	21741581	0	3.8E+07	41.28%	1.67%	57.05%	637385	21741581				
4-19F42P5_Sulcus_OSVZ_S14_out	13698111	493872	11594	19677503	0	3.4E+07	40.43%	1.49%	58.08%	505466	19677503				
5-19F44P5_Gyrus_OSVZ_S17_out	13911058	626766	20274	23278508	0	3.8E+07	36.77%	1.71%	61.52%	647040	23278508				
5-19F44P5_Sulcus_OSVZ_S18_out	15249050	532073	15456	26013512	0	4.2E+07	36.47%	1.31%	62.22%	547529	26013512				
6-19F17P15_Gyrus_OSVZ_S21_out	9674105	438337	8968	23194943	0	3.3E+07	29.04%	1.34%	69.62%	447305	23194943				
6-19F17P15_Sulcus_OSVZ_S22_out	11248083	509799	11338	27053093	0	3.9E+07	28.97%	1.34%	69.68%	521137	27053093				
7-19F18P15_Gyrus_OSVZ_S25_out	14068054	405955	21593	22080415	0	3.7E+07	38.46%	1.17%	60.37%	427548	22080415				
7-19F18P15_Sulcus_OSVZ_S26_out	13698293	369251	11138	21325331	0	3.5E+07	38.69%	1.07%	60.23%	380389	21325331				
8-19F19P15_Gyrus_OSVZ_S29_out	15053889	510969	12311	25552930	0	4.1E+07	36.60%	1.27%	62.13%	523280	25552930				
8-19F19P15_Sulcus_OSVZ_S30_out	14147215	457669	12247	23148745	0	3.8E+07	37.46%	1.24%	61.30%	469916	23148745				
9-19F20P15_Gyrus_OSVZ_S33_out	10967873	423535	9368	23060965	0	3.4E+07	31.83%	1.26%	66.92%	432903	23060965				
9-19F20P15_Sulcus_OSVZ_S34_out	11607950	486961	9961	23585394	3569	3.6E+07	32.52%	1.39%	66.09%	496922	23588963				

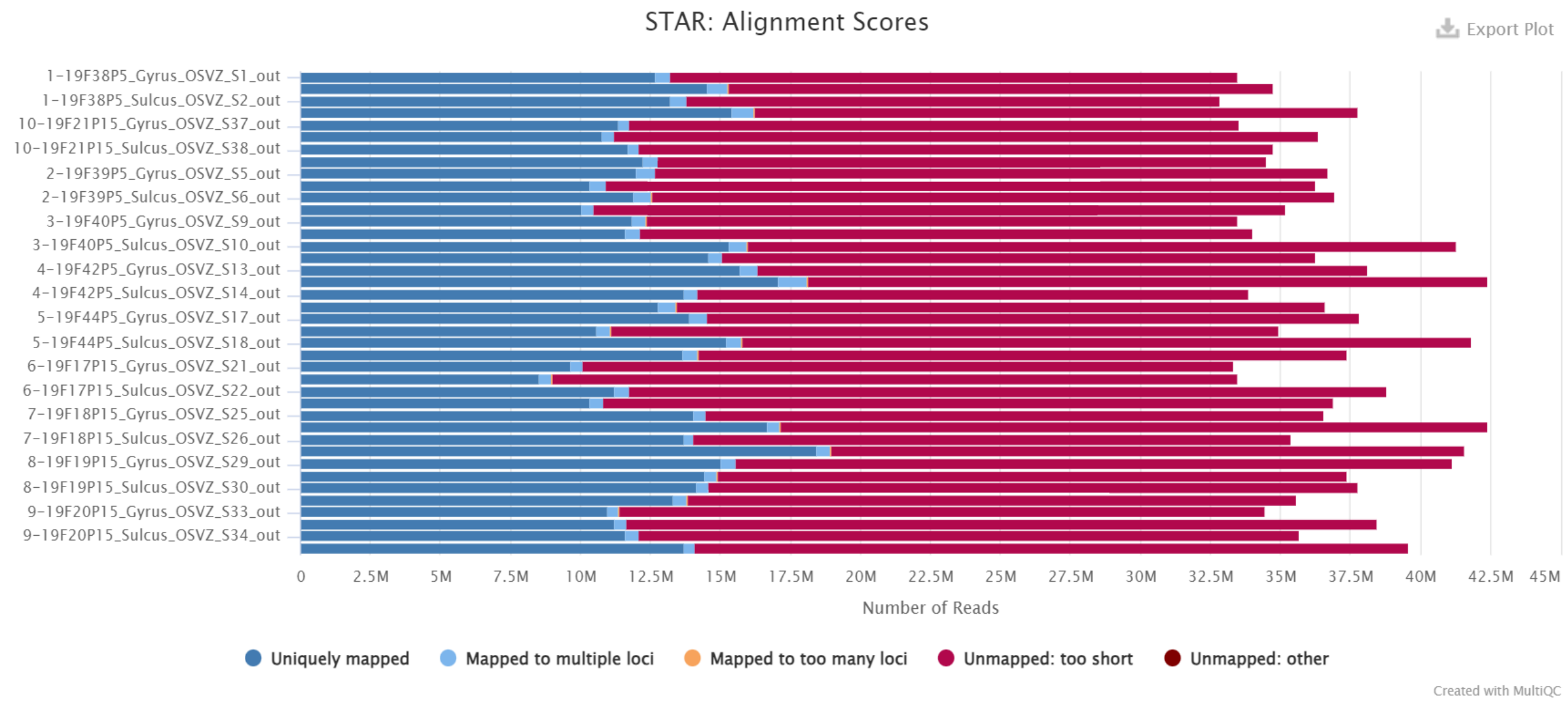

Sample/Animal Number (& Read)	Total Sequences	Sequences Flagged as Poor Quality	Sequence Length	%GC	Base Statistics Summary	Per Base Sequence Quality
19F38 P5 GYRUS R1	40029628	0	35-111	41	PASS	PASS
19F38 P5 GYRUS R2	40029628	0	35-111	43	PASS	PASS
19F38 P5 SULCUS R1	41080677	0	35-111	41	PASS	PASS
19F38 P5 SULCUS R2	41080677	0	35-111	43	PASS	PASS
19F39 P5 GYRUS R1	45320237	0	35-111	45	PASS	PASS
19F39 P5 GYRUS R2	45320237	0	63-111	46	PASS	PASS
2-19F39P5_SULCUS_OSVZ_R1	49766266	0	35-111	43	PASS	PASS
2-19F39P5_SULCUS_OSVZ_R2	49766266	0	35-111	44	PASS	PASS
3-19F40P5_GYRUS_OSVZ_R1	43818198	0	35-111	41	PASS	PASS
3-19F40P5_GYRUS_OSVZ_R2	43818198	0	87-111	43	PASS	PASS
3-19F40P5_SULCUS_OSVZ_R1	55781507	0	35-111	42	PASS	PASS
3-19F40P5_SULCUS_OSVZ_R2	55781507	0	61-111	44	PASS	PASS
4-19F42P5_GYRUS_OSVZ_R1	48175262	0	35-111	41	PASS	PASS
4-19F42P5_GYRUS_OSVZ_R2	48175262	0	35-111	43	PASS	PASS
4-19F42P5_SULCUS_OSVZ_R1	41262162	0	35-111	41	PASS	PASS
4-19F42P5_SULCUS_OSVZ_R2	41262162	0	35-111	43	PASS	PASS
5-19F44P5_GYRUS_OSVZ_R1	46342963	0	35-111	41	PASS	PASS
5-19F44P5_GYRUS_OSVZ_R2	46342963	0	35-111	43	PASS	PASS
5-19F44P5_SULCUS_OSVZ_R1	51398435	0	35-111	40	PASS	PASS
5-19F44P5_SULCUS_OSVZ_R2	51398435	0	69-111	42	PASS	PASS
6-19F17P15_GYRUS_OSVZ_R1	43219965	0	35-111	42	PASS	PASS
6-19F17P15_GYRUS_OSVZ_R2	43219965	0	82-111	43	PASS	PASS
6-19F17P15_SULCUS_OSVZ_R1	51434590	0	35-111	41	PASS	PASS
6-19F17P15_SULCUS_OSVZ_R2	51434590	0	35-111	43	PASS	PASS
7-19F18P15_GYRUS_OSVZ_R1	43962387	0	35-111	39	PASS	PASS
7-19F18P15_GYRUS_OSVZ_R2	43962387	0	81-111	40	PASS	PASS
7-19F18P15_SULCUS_OSVZ_R1	44618748	0	35-111	40	PASS	PASS
7-19F18P15_SULCUS_OSVZ_R2	44618748	0	84-111	43	PASS	PASS
8-19F19P15_GYRUS_OSVZ_R1	54664566	0	35-111	42	PASS	PASS
8-19F19P15_GYRUS_OSVZ_R2	54664566	0	83-111	43	PASS	PASS
8-19F19P15_SULCUS_OSVZ_R1	50156895	0	35-111	41	PASS	PASS
8-19F19P15_SULCUS_OSVZ_R2	50156895	0	35-111	43	PASS	PASS
9-19F20P15_GYRUS_OSVZ_R1	40037563	0	35-111	41	PASS	PASS
9-19F20P15_GYRUS_OSVZ_R2	40037563	0	39-111	43	PASS	PASS
9-19F20P15_SULCUS_OSVZ_R1	43436832	0	35-111	41	PASS	PASS
9-19F20P15_SULCUS_OSVZ_R2	43436832	0	59-111	42	PASS	PASS
10-19F21P15_GYRUS_OSVZ_R1	42329957	0	35-111	41	PASS	PASS
10-19F21P15_GYRUS_OSVZ_R2	42329957	0	35-111	42	PASS	PASS
10-19F21P15_SULCUS_OSVZ_R1	41629901	0	35-111	40	PASS	PASS
10-19F21P15_SULCUS_OSVZ_R2	41629901	0	35-111	42	PASS	PASS

Sample_ID	Assigned	Unassigned_Unmapped	Unassigned_Multimapping	Unassigned_NoFeatures	Unassigned_Ambiguous	Total Reads	Successfully Assigned Reads (%)	Multi-mapped Reads (%)	Unmapped Reads (%)	Average Successfully assigned reads
19F38 P5 GYRUS	2941880	20269607	1498735	9723982	32757	34466961	8.54%	4%	87%	8.49%
19F38 P5 SULCUS	2785162	19025899	1688422	10423633	28914	33952030	8.20%	5%	87%	0.94%
19F39 P5 GYRUS	3512054	24042473	1707633	8462365	47673	37772198	9.30%	5%	86%	
19F39 P5 SULCUS	3114306	24381670	1767764	8778307	33740	38075787	8.18%	5%	87%	
19F40 P5 GYRUS	3545754	21101395	1426462	8270844	39634	34384089	10.31%	4%	86%	
19F40 P5 SULCUS	4277326	25308735	1679074	11012459	45355	42322949	10.11%	4%	86%	
19F42 P5 GYRUS	3538070	21755421	1756808	12163058	33026	39246383	9.02%	4%	87%	
19F42 P5 SULCUS	2882230	19689097	1418358	10792124	25499	34807308	8.28%	4%	88%	
19F44 P5 GYRUS	3222928	23298782	1836303	10653440	36555	39048008	8.25%	5%	87%	
19F44 P5 SULCUS	3390621	26028968	1541597	11827301	35078	42823565	7.92%	4%	88%	
19F17 P15 GYRUS	3038492	23203911	1148199	6609415	28440	34028457	8.93%	3%	88%	
19F17 P15 SULCUS	3542289	27064431	1359772	7675474	33462	39675428	8.93%	3%	88%	
19F18 P15 GYRUS	2711283	22102008	1392363	11336848	21587	37564089	7.22%	4%	89%	
19F18 P15 SULCUS	2690062	21336469	1081503	10989851	20429	36118314	7.45%	3%	90%	
19F19 P15 GYRUS	4095208	25565241	1384348	10928504	35291	42008592	9.75%	3%	87%	
19F19 P15 SULCUS	3484161	23160992	1273050	10637859	29849	38585911	9.03%	3%	88%	
19F20 P15 GYRUS	2618470	23070333	1159780	8326796	23195	35198574	7.44%	3%	89%	
19F20 P15 SULCUS	2488477	23598924	1374775	9102905	18286	36583367	6.80%	4%	89%	
19F21 P15 GYRUS	2745985	21759640	1022953	8609398	23584	34161560	8.04%	3%	89%	
19F21 P15 SULCUS	2858303	22635892	1066676	8822911	25459	35409241	8.07%	3%	89%	

RNA Quality Control Summary Data for P5 and P15 Samples.

Age	Sample/ROI		RNA Concentration (pg/µl)	RIN	rRNA Ratio (28s/18s)	Read pairs assigned (per million)
P5	Animal 1	CG	623	5.8	0.5	40
		SSS	315	5.8	0.8	41.1
	Animal 2	CG	332	7.7	1.9	45.3
		SSS	373	7.4	1.5	49.8
	Animal 3	CG	2149	7.3	1.4	43.8
		SSS	974	6.8	1.5	55.8
	Animal 4	CG	655	7.3	0.9	48.2
		SSS	398	5.5	1.1	41.3
	Animal 5	CG	874	6.9	1.2	46.3
		SSS	791	7.2	1.1	51.4
P15	Animal 6	CG	910	6.9	1.3	43.2
		SSS	901	6.1	1.4	51.4
	Animal 7	CG	369	7.4	2.2	44
		SSS	378	6.3	0.7	44.6
	Animal 8	CG	405	8	1.4	54.7
		SSS	335	7	1.7	50.2
	Animal 9	CG	237	5.9	0.6	40
		SSS	312	6.8	0.9	43.4
	Animal 10	CG	332	7.7	0.8	42.3
		SSS	175	7.5	1	41.6

Note . rRNA ratios ≥ 1.0 are displayed in green. rRNA ratios < 1.0 are displayed in red. Abbreviations: bp, base pairs; P, postnatal day; RIN, RNA integrity number.
