# Supplementary material for: A transcriptional atlas of gyrification reveals dynamic spatio-temporal gene expression in the developing ferret cortex
Source: Brain Struct Funct. 2026 Aug 12;231(7):118. doi: 10.1007/s00429-026-03178-6 (PMC13469505; doi:10.1007/s00429-026-03178-6)
Supplement: Supplementary file 2 [file 429_2026_3178_MOESM2_ESM.pdf]

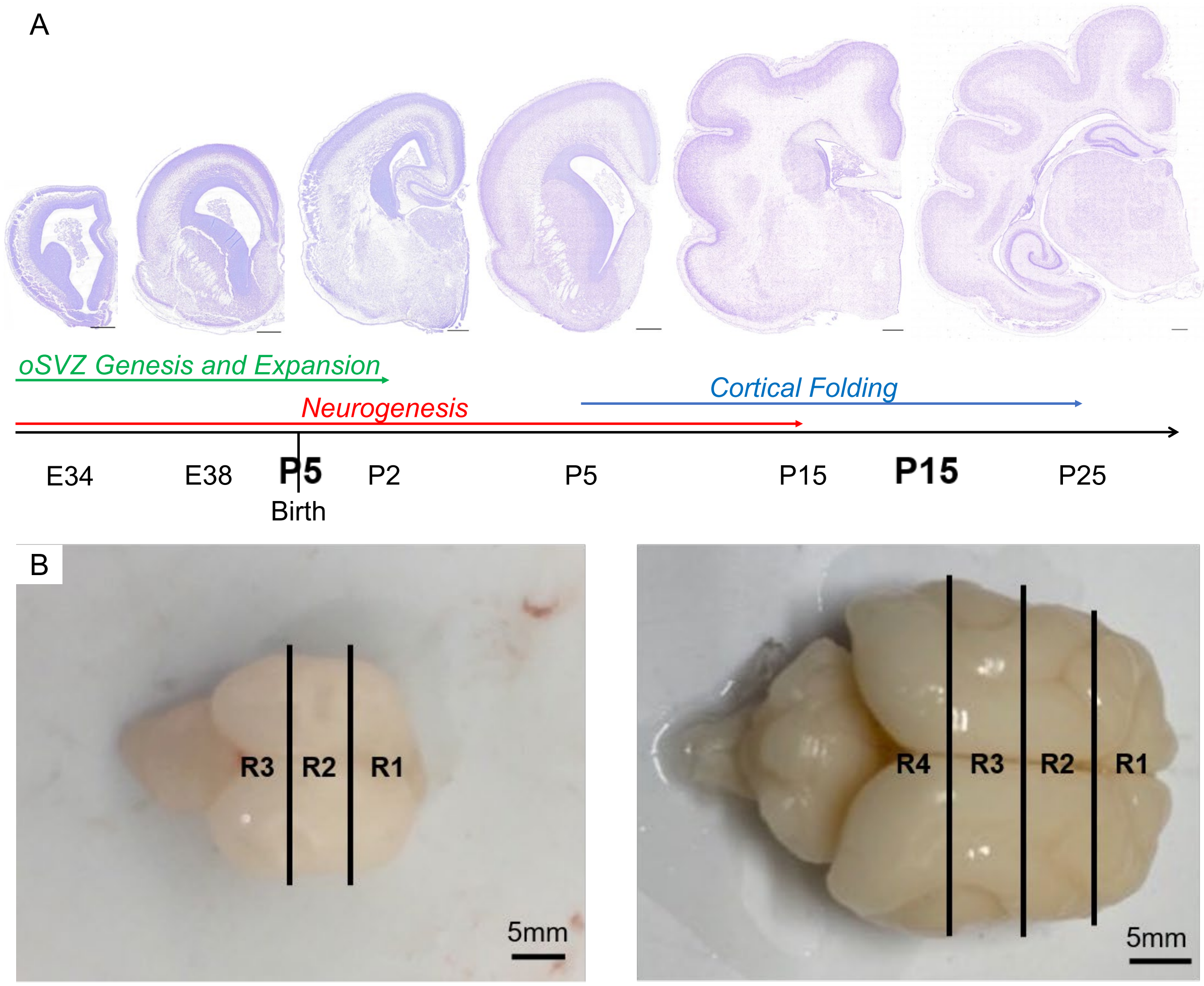

Supplementary Figure 1. Neurodevelopmental Timeline and Tissue Collection in the Ferret Brain. A. Nissl-stained coronal sections of the ferret brain at embryonic day 34 (E34) through to postnatal day 25 (P25) depicting the expansion and formation of cortical folds. The outer subventricular zone (oSVZ) forms at approximately E33 in the ferret brain and becomes the main source of upper layer neurons for the expanding cerebral cortex. The onset of gyrification occurs between 4 – 6 days after birth and is largely complete by around P28 (P25 shown here). Scale bars = 1mm. **B.** Tissue collection strategy for rostro-caudal regions in the ferret brain. Brains at P5 (left) and P15 (right) were extracted, hemisected, and subdivided into 5mm rostro-caudal regions: R1, R2, etc. Scale bars = 5mm.

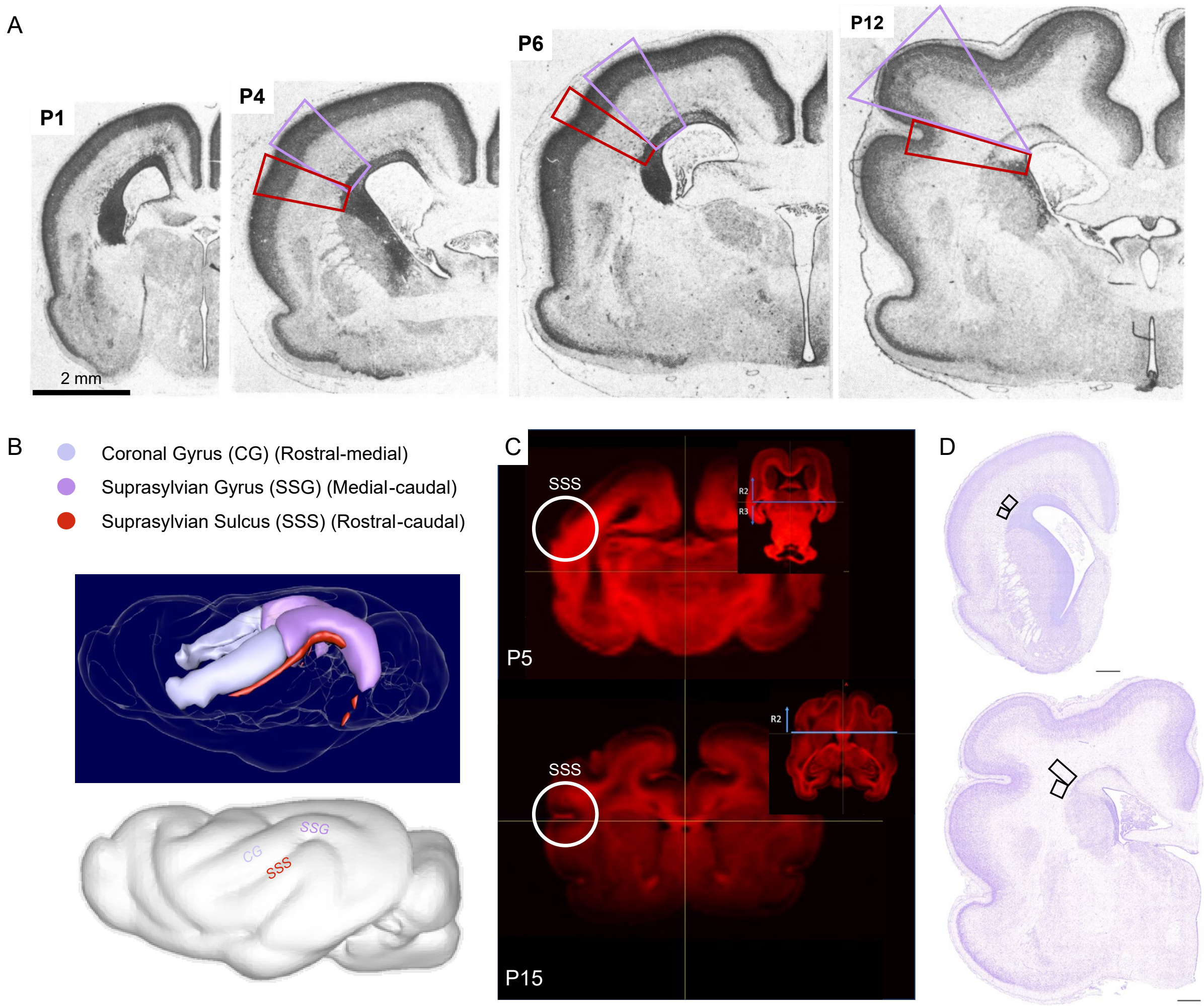

Supplementary Figure 2. Developmental and Structural Progression of Identifiable Gyri and Sulci in the Postnatal Ferret and Selection of Region of Interest. A. The coronal gyrus (CG) and suprasylvian sulcus (SSS) are the first discernible structures to develop in the postnatal ferret, visible from approximately postnatal day 4 (P4). Identifying this landmark sulcus allows for the intuitive identification of the CG situated immediately above it. Image adapted from Smart & McSherry, 1986a Scale bar = 2mm. **B.** Three-dimensional representation of selected regions of interest in the adult ferret. Adult structures are shown for illustrative purposes. During early postnatal ferret development, the CG and SSS are proportionately smaller and positioned more proximal to the cortical midline compared to the adult topography. As the cortex continues to develop into adulthood, these structures expand and shift outwards along the mediolateral axis to their final position. Images sourced from Bakker, Tiesinga, & Kötter, 2015, and Hutchinson et al., 2017. **C.** Magnetic resonance images (MRI) of P5 and P15 ferret brains illustrating SSS landmark. MRI scans of P5 and P15 ferrets (bottom panel). White circles indicate the approximate location of the SSS at both ages at the R2 level (5-10mm depth, see Figure 2.1). **D.** Representative images of the oSVZ beneath the CG and SSS at P5 and P15. Identification of the outer subventricular zone (oSVZ) beneath the (pre-)CG and (pre-)SSS at P5 and P15 for laser capture microdissection, corresponding with the CG and SSS of age-matched MRI and published reference images. To reliably identify and sample the oSVZ within the CG and SSS regions at P5, in-house anatomical selection criteria were developed. These criteria incorporated Hoechst staining to delineate cortical laminae, in combination with published anatomical references and established neuroanatomical landmarks. The cortical architecture described by Smart & McSherry (1986a; 1986b) from P1 to adulthood was used to confirm the position of key gyri and sulci in sections near the interventricular foramen, corresponding approximately to regions R2 through the early portion of R3 in our P5 and P15 samples. These regions were further verified by the presence of large cerebral ventricles. To aid in accurate oSVZ identification, published reference images of the germinal zones in the developing ferret cortex were used as a visual guide (See Bilgic et al. (2023) Supplementary Figure 1A and 1B; de Juan Romero et al. (2015) Figures 1C and 1E, 2D, 5A, and 6; Reillo et al. (2011) Figures 1A, and 3F-L; Reillo and Borrell

(2012) Figures 1, 4, and 7). At P5, the oSVZ follows the contour of the ventricle, thickening in gyral regions. At P15, the oSVZ disperses into the largely expanded gyri, becoming much more cell sparse. Scale bar = 1mm. Abbreviations: CS, coronal sulcus; SSG, suprasylvian gyrus.

Supplementary Tables

Supplementary Table 1. GO Analysis of DEGs between the CG oSVZ and the SSS oSVZ at P5 (absolute logFC > 2, *p* < .05).

GO ID	Description	*p* Value	*FDR Adj. p Value*	Gene ID
GO:0002576	Platelet degranulation	0.0000131061	0.032896421	*CYB5R1/CLU/TUBA4A/LYN/HGF/PDGFA/CTSW/VEGFB*
GO:1905039	Carboxylic acid transmembrane transport	0.001665802	0.617663573	*ATP1A2/SLC7A10/SLC16A2/SLC25A11/SLC38A5/SLC25A1*
GO:1903825	Organic acid transmembrane transport	0.001722947	0.617663573	*ATP1A2/SLC7A10/SLC16A2/SLC25A11/SLC38A5/SLC25A1*
GO:0071539	Protein localization to centrosome	0.002180973	0.617663573	*SPAG5/KIAA0753/PARD6A*
GO:0030178	Negative regulation of Wnt signalling pathway	0.002508273	0.617663573	*PSMA7/GLI1/NPHP4/LRP4/FZD1/UBAC2/TAX1BP3*
GO:1905508	Protein localization to microtubule organizing center	0.002615073	0.617663573	*SPAG5/KIAA0753/PARD6A*
GO:0048875	Chemical homeostasis within a tissue	0.0036478	0.617663573	*NAPSA/LPCAT1*
GO:0001825	Blastocyst formation	0.004222793	0.617663573	*FURIN/MFN2/NLE1*
GO:0035845	Photoreceptor cell outer segment organization	0.00435337	0.617663573	*NPHP4/MFSD2A*
GO:0090090	Negative regulation of canonical Wnt signalling pathway	0.004589531	0.617663573	*PSMA7/GLI1/NPHP4/LRP4/FZD1/UBAC2*

Abbreviations: CG, coronal gyrus; DEG, differentially expressed gene; FDR, false discovery rate; GO, gene ontology; oSVZ, outer subventricular zone; P, postnatal day; SSS, suprasylvian sulcus.

Supplementary Table 2. KEGG Pathway Analysis of DEGs between the CG oSVZ and the SSS oSVZ at P5 (absolute logFC > 2, *p* < .05).

ID	Description	*p* Value	*FDR Adj. p Value*	Gene ID
hsa00562	Inositol phosphate metabolism	0.004117617	0.448090307	*PI4KB/PLCB3/MTMR1/ALDH6A1*
hsa00230	Purine metabolism	0.005776649	0.448090307	*NTPCR/NME4/PGM2/PDE3A/AK5*
hsa00730	Thiamine metabolism	0.007776854	0.448090307	*NTPCR/AK5*
hsa00520	Amino sugar and nucleotide sugar metabolism	0.009583538	0.448090307	*CYB5R1/PGM2/GNPDA1*
hsa04015	Rap1 signalling pathway	0.011315412	0.448090307	*RASGRP2/HGF/PDGFA/PLCB3/VEGFB/PARD6A*
hsa04010	MAPK signalling pathway	0.016181504	0.533989628	*PPP3R1/RASGRP2/CACNB4/HGF/RPS6KA4/PDGFA/VEGFB*
hsa04020	Calcium signalling pathway	0.020622315	0.583316917	*PPP3R1/HGF/PDGFA/CAMK1D/PLCB3/VEGFB*
hsa04924	Renin secretion	0.023994145	0.593855094	*PPP3R1/PLCB3/PDE3A*
hsa04142	Lysosome	0.03079677	0.621713575	*NAPSA/AP3B2/CTSO/CTSW*
hsa00410	Beta-alanine metabolism	0.031399675	0.621713575	*SMOX/ALDH6A1*

Abbreviations: CG, coronal gyrus; DEG, differentially expressed gene; FDR, false discovery rate; GO, gene ontology; oSVZ, outer subventricular zone; P, postnatal day; SSS, suprasylvian sulcus.

Supplementary Table 3. GO Analysis of DEGs between the CG oSVZ and the SSS oSVZ at P15 (absolute logFC > 2, $p < .05$).

GO ID	Description	*p* Value	*FDR Adj. p Value*	Gene ID
GO:0001837	Epithelial to mesenchymal transition	0.000348448	0.219267065	*EOMES/TMEM100/BCL9L/AKNA/SMAD3*
GO:0031103	Axon regeneration	0.00115945	0.219267065	*SPP1/MAG/GRN*
GO:0031953	Negative regulation of protein autophosphorylation	0.001182301	0.219267065	*GFRA2/ENPP1*
GO:1901215	Negative regulation of neuron death	0.001558912	0.219267065	*TMEM259/MAG/SERPINF1/GABRA5/GRN*
GO:0031102	Neuron projection regeneration	0.001671927	0.219267065	*SPP1/MAG/GRN*
GO:0090336	Positive regulation of brown fat cell differentiation	0.002040332	0.219267065	*PRDM16/RREB1*
GO:0045453	Bone resorption	0.00211254	0.219267065	*SPP1/IAPP/NOX4*
GO:0048762	Mesenchymal cell differentiation	0.002183961	0.219267065	*EOMES/TMEM100/BCL9L/AKNA/SMAD3*
GO:0007040	Lysosome organization	0.002408382	0.219267065	*VPS18/RAB34/GRN*
GO:0080171	Lytic vacuole organization	0.002408382	0.219267065	*VPS18/RAB34/GRN*

Abbreviations: CG, coronal gyrus; DEG, differentially expressed gene; FDR, false discovery rate; GO, gene ontology; oSVZ, outer subventricular zone; P, postnatal day; SSS, suprasylvian sulcus.

Supplementary Table 4. KEGG Pathway Analysis of DEGs between the CG oSVZ and the SSS oSVZ at P15 (absolute logFC > 2, *p* < .05).

ID	Description	*p* Value	*FDR Adj. p Value*	Gene ID
hsa05212	Pancreatic cancer	0.003103924	0.281591934	*RALGDS/SMAD3/E2F3*
hsa05033	Nicotine addiction	0.010436676	0.281591934	*GABRG1/GABRA5*
hsa04080	Neuroactive ligand-receptor interaction	0.012836906	0.281591934	*GABRG1/RXFP2/IAPP/GABRA5/NMB*
hsa03022	Basal transcription factors	0.013087599	0.281591934	*ERCC2/GTF2H5*
hsa03420	Nucleotide excision repair	0.014221815	0.281591934	*ERCC2/GTF2H5*
hsa05220	Chronic myeloid leukemia	0.03497022	0.498840513	*SMAD3/E2F3*
hsa05210	Colorectal cancer	0.043787355	0.498840513	*RALGDS/SMAD3*
hsa04727	Gabaergic synapse	0.046579113	0.498840513	*GABRG1/GABRA5*
hsa05032	Morphine addiction	0.048476191	0.498840513	*GABRG1/GABRA5*
hsa04933	AGE-RAGE signalling pathway in diabetic complications	0.057353439	0.498840513	*NOX4/SMAD3*

Abbreviations: CG, coronal gyrus; DEG, differentially expressed gene; FDR, false discovery rate; GO, gene ontology; oSVZ, outer subventricular zone; P, postnatal day; SSS, suprasylvian sulcus.

Supplementary Table 5. GO Analysis of DEGs between the CG oSVZ at P5 compared to P15 (absolute logFC > 2, *FDR* < .01).

GO ID	Description	*p* Value	*FDR Adj. p Value*	Gene ID
GO:0007411	Axon guidance	3.99872E-20	$9.79955e^{-17}$	*SEMA3C/EPHA3/TBR1/ROBO1/ALCAM/SEMA3E/ROBO2/TUBB3/FOXG1/SPTBN1/KIF5A/SEMA6A/KIF5C/LRP1/NRP2/EPHA5/SEMA5B/PTPRM/L1CAM/SEMA5A/DOK5/DLX5/FEZ2/DAG1/VEGFA/NFASC/EFNB1/UNC5D/ANOS1/LAMA1/LAMB2/CRMP1/B3GNT2/TUBB2B/CSF1R/LGI1/PRKCQ/CXCR4/SEMA3D/TNR*
GO:0097485	Neuron projection guidance	4.55475E-20	$9.79955e^{-17}$	*SEMA3C/EPHA3/TBR1/ROBO1/ALCAM/SEMA3E/ROBO2/TUBB3/FOXG1/SPTBN1/KIF5A/SEMA6A/KIF5C/LRP1/NRP2/EPHA5/SEMA5B/PTPRM/L1CAM/SEMA5A/DOK5/DLX5/FEZ2/DAG1/VEGFA/NFASC/EFNB1/UNC5D/ANOS1/LAMA1/LAMB2/CRMP1/B3GNT2/TUBB2B/CSF1R/LGI1/PRKCQ/CXCR4/SEMA3D/TNR*
GO:0007409	Axonogenesis	9.36674E-20	$1.3435e^{-16}$	*SEMA3C/EPHA3/TBR1/NTRK2/STMN1/ROBO1/ALCAM/APOE/OMG/SEMA3E/ROBO2/TUBB3/UST/FOXG1/SPTBN1/KIF5A/SEMA6A/KIAA0319/KIF5C/LRP1/MAP1A/ISLR2/NRP2/EPHA5/SEMA5B/LRRC4C/PTPRM/L1CAM/SEMA5A/DOK5/DLX5/FN1/FEZ2/DAG1/VEGFA/NFASC/POU3F2/EFNB1/UNC5D/ANOS1/LAMA1/LAMB2/CRMP1/B3GNT2/TUBB2B/CSF1R/LGI1/PRKC*

				Q/CXCR4/SEMA3D/TNR
GO:0022604	Regulation of cell morphogenesis	5.56088E-13	$5.98211e^{-10}$	*SEMA3C/TBR1/CD44/NTRK2/ROBO1/APOE/POSTN/EEF2K/CSPG5/OMG/SEMA3E/ROBO2/UST/CUX2/LPAR1/SEMA6A/KIAA0319/LRP1/ENPP2/ISLR2/SEMA5B/LRRC4C/L1CAM/SEMA5A/KANK1/FN1/PDZD8/DAG1/VEGFA/LIMS2/ARHGAP15/POU3F2/KIF1A/MYO10/TUBB2B/EPB41L3/CSF1R/ITGA7/LIMS1/CXCR4/SEMA3D/TNR*
GO:0034329	Cell junction assembly	2.27465E-12	$1.82597e^{-09}$	*APC/EPHA3/NTRK2/EEF2K/ROBO2/CUX2/DST/CD9/UGT8/LRP1/TJP1/CDH20/CLDN11/PTPRK/ITGA6/TLN2/PEAK1/APOD/FAM107A/LIMCH1/LRRTM3/CDH9/FN1/STRN/VEGFA/NFASC/LIMS2/FMN1/GRID2/GJA1/LAMC1/CLSTN2/EPB41L3/LIMS1/ITGA2/CDH7/ADD2/IKBKB*
GO:0030198	Extracellular matrix organization	2.75837E-12	$1.82597e^{-09}$	*CD44/ADAMTS9/POSTN/VCAN/HAPLN1/HTRA1/PDGFRA/VCAM1/SULF1/CST3/COL11A1/LRP1/ITGB8/ITGA6/LOX/PTX3/ECM2/LAMA4/FN1/ADAMTS18/DAG1/COL9A2/ADAMTS6/FBLN2/LAMA1/LCP1/LAMB2/CTSS/COL4A4/PDGFA/LAMC1/CCDC80/ITGA7/ITGA2/FAP/TNR*
GO:0043062	Extracellular structure organization	2.97044E-12	$1.82597e^{-09}$	*CD44/ADAMTS9/POSTN/VCAN/HAPLN1/HTRA1/PDGFRA/VCAM1/SULF1/CST3/COL11A1/LRP1/ITGB8/ITGA6/LOX/PTX3/ECM2/LAMA4/FN1/ADAMTS18/DAG1/COL9A2/ADAMTS6/FBLN2/LAMA1/LCP1/*

				LAMB2/CTSS/COL4A4/PDGFA/LAMC1/CCDC80/ITGA7/ITGA2/FAP/TNR
GO:0010769	Regulation of cell morphogenesis involved in differentiation	8.87335E-12	4.77276e^{-09}	*SEMA3C/TBR1/NTRK2/ROBO1/APOE/POSTN/EEF2K/CSPG5/OMG/SEMA3E/ROBO2/UST/CUX2/SEMA6A/KIAA0319/LRP1/ISLR2/SEMA5B/LRRC4C/L1CAM/SEMA5A/KANK1/FN1/VEGFA/LIMS2/POU3F2/KIF1A/TUBB2B/LIMS1/SEMA3D/TNR*
GO:0050770	Regulation of axonogenesis	6.6486E-11	3.17877e^{-08}	*SEMA3C/TBR1/NTRK2/ROBO1/APOE/OMG/SEMA3E/ROBO2/UST/SEMA6A/KIAA0319/LRP1/ISLR2/SEMA5B/LRRC4C/L1CAM/SEMA5A/FN1/VEGFA/POU3F2/TUBB2B/SEMA3D/TNR*
GO:0031589	Cell-substrate adhesion	3.67154E-10	1.57986e^{-07}	*ANGPT1/EPHA3/CD44/ADAMTS9/POSTN/CSPG5/SEMA3E/VCAM1/LRP1/PTPRK/L1CAM/ITGA6/PEAK1/APOD/ECM2/FAM107A/LIMCH1/KANK1/FN1/DAG1/VEGFA/LIMS2/FMN1/FBLN2/LAMB2/LAMC1/CCDC80/ITGA7/LIMS1/ITGA2/RIN2*

Abbreviations: CG, coronal gyrus; DEG, differentially expressed gene; FDR, false discovery rate; GO, gene ontology; oSVZ, outer subventricular zone; P, postnatal day.

Supplementary Table 6. KEGG Pathway analysis of DEGs between the CG oSVZ at P5 compared to P15 (absolute logFC > 2, *FDR* < .01).

ID	Description	*p* Value	*FDR Adj. p Value*	Gene ID
hsa04512	ECM-receptor interaction	4.76379E-09	$1.18142e^{-06}$	*CD44/ITGB8/FRAS1/ITGA6/LAMA4/FN1/DAG1/COL9A2/LAMA1/LAMB2/COL4A4/LAMC1/ITGA7/ITGA2/TNR*
hsa04151	PI3K-Akt signalling pathway	7.97222E-08	$9.88556e^{-06}$	*ANGPT1/NTRK2/PDGFRA/LPAR1/FLT1/ITGB8/PDGFC/SGK1/ITGA6/LAMA4/FN1/GNB4/IL7R/VEGFA/ERBB3/FGF1/COL9A2/LAMA1/LAMB2/COL4A4/CREB5/PDGFA/LAMC1/CSF1R/ITGA7/ITGA2/TNR/IKBKB*
hsa04510	Focal adhesion	3.93621E-06	0.000325393	*PDGFRA/FLT1/ITGB8/PDGFC/ITGA6/TLN2/LAMA4/FN1/VEGFA/COL9A2/LAMA1/LAMB2/COL4A4/PDGFA/LAMC1/ITGA7/ITGA2/TNR*
hsa04360	Axon guidance	1.53964E-05	0.000954579	*SEMA3C/EPHA3/ROBO1/SEMA3E/ROBO2/SEMA6A/EPHA5/SEMA5B/LRRC4C/L1CAM/SEMA5A/EFNB1/UNC5D/SSH2/CXCR4/SEMA3D*
hsa04810	Regulation of actin cytoskeleton	2.09711E-05	0.001040166	*APC/PDGFRA/SCIN/LPAR1/ITGB8/PDGFC/ITGA6/NCKAP1L/CYFIP2/FN1/ARHGEF6/FGF1/WASF2/SSH2/PDGFA/ITGA7/ITGA2/CXCR4*
hsa04540	Gap junction	7.56468E-05	0.003126735	*PDGFRA/TUBB3/LPAR1/TUBB/ITPR2/TJP1/PDGFC/GJA1/PDGFA/TUBB2B*
hsa05412	Arrhythmogenic right ventricular cardiomyopathy	0.000139388	0.004938328	*TCF7L2/ITGB8/ITGA6/DAG1/RYR2/LAMA1/GJA1/ITGA7/ITGA2*

hsa05165	Human papillomavirus infection	0.000293459	0.009097235	*APC/ATP6V0A2/MAML2/TCF7L2/ITGB8/ITGA6/LAMA4/FN1/VEGFA/COL9A2/LAMA1/LAMB2/COL4A4/CREB5/LAMC1/ITGA7/ITGA2/TNR/HEYL/IKBKB*
hsa05222	Small cell lung cancer	0.000539562	0.014867926	*ITGA6/LAMA4/FN1/LAMA1/LAMB2/COL4A4/LAMC1/ITGA2/IKBKB*
hsa04724	Glutamatergic synapse	0.000641156	0.014938809	*GRIA4/ITPR2/SLC1A1/GRIA3/GRIK1/GNB4/SLC17A6/PLD1/GRM3/SLC1A2*

Abbreviations: CG, coronal gyrus; DEG, differentially expressed gene; FDR, false discovery rate; oSVZ, outer subventricular zone; P, postnatal day.

Supplementary Table 7. GO Analysis of DEGs between the SSS oSVZ at P5 compared to P15 (absolute logFC > 2, *FDR* < .01).

GO ID	Description	*p* Value	*FDR Adj. p Value*	Gene ID
GO:0007411	Axon guidance	8.30886E-18	$2.10166e^{-14}$	*SEMA3C/EPHA3/ROBO1/TBR1/SEMA3E/ROBO2/SPTBN1/ALCAM/SEMA6A/KIF5C/TUBB3/EPHA5/FOXG1/LRP1/NRP2/FYN/DAG1/TMEFF1/L1CAM/KIF5A/SEMA5A/SLIT2/DOK6/DOK5/LAMA2/CDH4/SEMA3A/DCC/UNC5D/PLXNA4/PLXNA2/PRKCA/PTCH1/ANOS1/CELSR3/DPYSL5/LAMB2/DLX5/CDK5R1*
GO:0097485	Neuron projection guidance	9.40339E-18	$2.10166e^{-14}$	*SEMA3C/EPHA3/ROBO1/TBR1/SEMA3E/ROBO2/SPTBN1/ALCAM/SEMA6A/KIF5C/TUBB3/EPHA5/FOXG1/LRP1/NRP2/FYN/DAG1/TMEFF1/L1CAM/KIF5A/SEMA5A/SLIT2/DOK6/DOK5/LAMA2/CDH4/SEMA3A/DCC/UNC5D/PLXNA4/PLXNA2/PRKCA/PTCH1/ANOS1/CELSR3/DPYSL5/LAMB2/DLX5/CDK5R1*
GO:0007409	Axonogenesis	2.93748E-17	$4.37685e^{-14}$	*SEMA3C/EPHA3/ROBO1/APOE/STMN1/NTRK2/TBR1/SEMA3E/ROBO2/SPTBN1/ALCAM/OMG/SEMA6A/KIF5C/TUBB3/EPHA5/UST/FOXG1/LRP1/NRP2/MAP1A/FYN/DAG1/LRRC4C/TMEFF1/L1CAM/KIF5A/SEMA5A/SLIT2/FN1/DOK6/DOK5/LAMA2/TIAM2/CDH4/SEMA3A/DCC/UNC5D/PLXNA4/PLXNA2/LRP4/DRD2/PRKCA/PTCH1/ANOS1/CELSR3/DPYSL5/LAMB2/DLX5/CDK5R1*
GO:0050769	Positive regulation of neurogenesis	1.71494E-14	$1.91645e^{-11}$	*EPHA3/ROBO1/APOE/CNTN1/NTRK2/ROBO2/LPAR1/CUX2/Z*

				NF365/ENC1/SEMA6A/RIMS2/IL6ST/FOXG1/LRP1/FYN/DAG1/L1CAM/BEND6/SEMA5A/SLIT2/FN1/SERPINE2/BIN1/PRKD1/EEF2K/TIAM2/ARSB/TIMP2/GPRC5B/CDH4/SEMA3A/SOX8/ITGA6/P2RY12/ZNF804A/PLXNA4/REST/PLXNA2/DRD2/NEGR1/NKX2-2/BMP2/RAPGEF1/SERPINF1/NEUROD1
GO:0022604	Regulation of cell morphogenesis	1.2847E-11	$1.06067e^{-08}$	*SEMA3C/CD44/CSPG5/ROBO1/APOE/POSTN/NTRK2/TBR1/SEMA3E/ROBO2/LPAR1/CUX2/OMG/SEMA6A/ENPP2/RIMS2/KANK1/UST/LRP1/FYN/DAG1/LRRC4C/LIMS2/L1CAM/SEMA5A/SLIT2/FN1/EEF2K/TIAM2/EPB41L3/CDH4/SEMA3A/DCC/PLXNA4/PLXNA2/LRP4/ITGA7/RND3/MYO10/ATP10A/CDK5R1/DLC1*
GO:0045666	Positive regulation of neuron differentiation	1.42372E-11	$1.06067e^{-08}$	*EPHA3/ROBO1/APOE/CNTN1/NTRK2/ROBO2/LPAR1/CUX2/ENC1/RIMS2/FOXG1/LRP1/FYN/L1CAM/BEND6/SEMA5A/SLIT2/FN1/PRKD1/EEF2K/TIAM2/ARSB/TIMP2/GPRC5B/CDH4/ITGA6/ZNF804A/PLXNA4/REST/PLXNA2/NEGR1/NKX2-2/BMP2/RAPGEF1/SERPINF1/NEUROD1*
GO:0034329	Cell junction assembly	4.00457E-11	$2.06931e^{-08}$	*APC/EPHA3/NTRK2/ROBO2/TJP1/CUX2/PKP4/LRRN3/DST/LRP1/UGT8/LRRTM3/APOD/FAM107A/CD9/LIMS2/FN1/PEAK1/ITGA2/CLDN11/EEF2K/LAMC1/NRG1/TLN2/GABRG2/EPB41L3/STRN/ITGA6/GJA1/LRP4/DRD2/PRKCA/GJB6/IL1RAP/RAP*

				GEF1/DLC1/RUNX1/SPOCK2
GO:0030198	Extracellular matrix organization	4.30884E-11	2.06931e^{-08}	*VCAN/CD44/POSTN/VCAM1/ADAMTS9/CST3/HAPLN1/PDGFRA/HTRA1/PTX3/SULF1/COL11A1/LRP1/BCAN/DAG1/ECM2/FN1/ITGA2/LAMC1/ADAMTS8/FBN1/COL9A2/ADAMTS6/LAMA2/TIMP2/ADAMTS18/CTSS/ITGA6/LOX/CRISPLD2/ITGA7/FMOD/LAMB2/NID1/P4HA1/SPOCK2*
GO:0010001	Glial cell differentiation	4.40081E-11	2.06931e^{-08}	*CSPG5/NTRK2/DAAM2/LPAR1/ZNF365/SOX6/IL6ST/LRP1/DAG1/CD9/SERPINE2/BIN1/NRG1/PLP1/LAMA2/STAT3/CLU/SOX8/ERBB3/SOX4/SIRT2/NKX2-2/BMP2/GPR17/GPR37L1/FA2H/LAMB2*
GO:0043062	Extracellular structure organization	4.62934E-11	2.06931e^{-08}	*VCAN/CD44/POSTN/VCAM1/ADAMTS9/CST3/HAPLN1/PDGFRA/HTRA1/PTX3/SULF1/COL11A1/LRP1/BCAN/DAG1/ECM2/FN1/ITGA2/LAMC1/ADAMTS8/FBN1/COL9A2/ADAMTS6/LAMA2/TIMP2/ADAMTS18/CTSS/ITGA6/LOX/CRISPLD2/ITGA7/FMOD/LAMB2/NID1/P4HA1/SPOCK2*

Abbreviations: DEG, differentially expressed gene; FDR, false discovery rate; GO, gene ontology; oSVZ, outer subventricular zone; P, postnatal day.

Supplementary Table 8. KEGG Pathway Analysis of DEGs between the CG oSVZ at P5 compared to P15 (absolute logFC > 2, *FDR* < .01).

ID	Description	*p* Value	*FDR Adj. p Value*	Gene ID
hsa04360	Axon guidance	9.51282E-09	2.42577e-06	*SEMA3C/EPHA3/ROBO1/SEMA3E/ROBO2/SEMA6A/EPHA5/FYN/LRRC4C/L1CAM/SEMA5A/SLIT2/ABLIM2/ABLIM3/SEMA3A/DCC/UNC5D/PLXNA4/PLXNA2/PRKCA/PTCH1/DPYSL5*
hsa04512	ECM-receptor interaction	7.03247E-06	0.00089664	*CD44/FRAS1/DAG1/FN1/ITGA2/LAMC1/CHAD/COL9A2/LAMA2/ITGA6/ITGA7/LAMB2*
hsa04540	Gap junction	3.92934E-05	0.003339939	*PDGFRA/LPAR1/TJP1/ITPR2/TUBB3/GJA1/PDGFC/DRD2/PRKCA/PLCB2/PRKG2*
hsa04724	Glutamatergic synapse	9.78461E-05	0.006237688	*GRIA4/ITPR2/SLC17A6/GRIA3/SLC1A1/GRIK1/GNB4/GRM3/PLD1/PRKCA/PLCB2/SLC17A7*
hsa05412	Arrhythmogenic right ventricular cardiomyopathy	0.000334226	0.017045549	*TCF7L2/DAG1/ITGA2/LAMA2/ITGA6/GJA1/ITGA7/RYR2/TCF7L1*
hsa04151	PI3K-Akt signalling pathway	0.000510919	0.021714041	*ANGPT1/NTRK2/PDGFRA/LPAR1/FGF1/FN1/ITGA2/SGK1/LAMC1/ANGPT2/CHAD/COL9A2/LAMA2/IL7R/KITLG/GNB4/ERBB3/ITGA6/PDGFC/ITGA7/PRKCA/LAMB2*
hsa04510	Focal adhesion	0.000719426	0.026207666	*PDGFRA/FYN/FN1/ITGA2/LAMC1/TLN2/CHAD/COL9A2/LAMA2/ITGA6/PDGFC/ITGA7/PRKCA/RAPGEF1/LAMB2*
hsa04514	Cell adhesion molecules	0.001820737	0.058035991	*VCAN/CNTN1/VCAM1/ALCAM/NCAM2/LRRC4C/L1CAM/CLDN11/CADM1/CDH4/ITGA6/NEGR1*

hsa04015	Rap1 signalling pathway	0.00284107	0.080496986	*ANGPT1/PDGFRA/LPAR1/FGF1/PRKD1/ANGPT2/TLN2/KITLG/APBB1IP/PDGFC/DRD2/PRKCA/PLCB2/RAPGEF1*
hsa04072	Phospholipase D signalling pathway	0.003472991	0.088561273	*PDGFRA/LPAR1/FYN/DGKB/KITLG/GRM3/PLD1/PDGFC/PRKCA/PLCB2/CYTH2*

Abbreviations: DEG, differentially expressed gene; FDR, false discovery rate; oSVZ, outer subventricular zone; P, postnatal day; SSS, suprasylvian sulcus.

Supplementary Table 9. Independent Samples T-Tests using Normalised RT-qPCR Data between Regions of Interest.

		P5 CG → SSS		
Gene	Relative FC	*t*	*p*	95% CI
SOX9	1.03	-.08	.96	[-1.28, 1.22]
PI4KB	1.47	-1.40	.20	[-1.25, 0.31]
FOXC1	0.73	0.43	.68	[-1.22, 1.76]
AJAP1	0.53	3.17	.01*	[0.13, 0.81]
PRUNE1	0.79	0.56	.59	[-0.68, 1.11]
SEMA3D	0.13	1.01	.39	[-1.88, 3.63]
FOXM1	0.58	1.65	.17	[-0.27, 1.12]
		P15 CG → SSS		
Gene	Relative FC	*t*	*p*	95% CI
SOX9	1.25	-0.83	.43	[-0.93, 0.44]
PI4KB	0.75	1.00	.35	[-0.32, 0.82]
FOXC1	1.24	-0.90	.40	[-0.84, 0.37]
AJAP1	1.23	-0.48	.65	[-1.39, 0.93]
PRUNE1	1.29	-0.55	.60	[-1.55, 0.96]
SEMA3D	0.95	0.53	.62	[-0.19, 0.30]
FOXM1	0.91	.31	.77	[-0.60, 0.78]

Note. Fold Change (FC) calculated as a ratio of the initial condition. As FC approaches 1.00, the magnitude of change in expression between groups is 0 (i.e. expression ratio is 1:1 in comparison groups). * $p < .05$. Abbreviations: CG, coronal gyrus; P, postnatal day; RT-qPCR, quantitative reverse transcription polymerase chain reaction; SSS; suprasylvian sulcus.

Supplementary Table 10. Independent Samples T-Tests using Normalised RT-qPCR Data for Age-wise Comparisons.

		P5 CG → P15 CG		
Gene	Relative FC	*t*	*p*	95% CI
SOX9	2.44	-2.04	.08	[-3.07, 0.19]
PI4KB	1.47	-1.06	.32	[-1.48, 0.55]
FOXC1	0.89	0.18	.86	[-1.29, 1.51]
AJAP1	0.46	2.47	.04*	[0.04, 1.05]
PRUNE1	1.00	-0.01	0.99	[-0.97, 0.97]
SEMA3D	0.26	0.86	0.45	[-2.01, 3.50]
FOXM1	1.82	-1.87	0.10	[-1.83, 0.19]
		P5 SSS → P15 SSS		
Gene	Relative FC	*t*	*p*	95% CI
SOX9	2.95	-3.56	.01**	[-3.21, -0.68]
PI4KB	0.75	1.60	.15	[-0.11, 0.61]
FOXC1	1.51	-1.24	.25	[-1.46, 0.44]
AJAP1	1.07	-0.27	.79	[0.65, 0.52]
PRUNE1	1.65	-1.03	.34	[-2.14, 0.85]
SEMA3D	1.90	-3.08	.03*	[-1.64, -0.16]
FOXM1	2.93	-2.40	.05*	[-3.83, -0.03]

Note. Fold Change (FC) calculated as a ratio of the initial condition. As FC approaches 1.00, the magnitude of change in expression between groups is 0 (i.e. expression ratio is 1:1 in comparison groups). * $p < .05$. ** $p < .01$. Abbreviations: CG, coronal gyrus; P, postnatal day; RT-qPCR, quantitative reverse transcription polymerase chain reaction; SSS; suprasylvian sulcus.
